# A Bayesian Bernoulli-Exponential joint model for binary longitudinal outcomes and informative time with applications to bladder cancer recurrence data

**DOI:** 10.1186/s12874-024-02160-2

**Published:** 2024-03-01

**Authors:** Michael Safo Oduro

**Affiliations:** 1 Pfizer Research & Development, PSSM Data Sciences, 445 Eastern Point Rd, Groton, Connecticut USA; 2 Department of Applied Statistics and Research Methods at the University of Northern Colorado, Greeley, Colorado, USA

**Keywords:** Joint modelling, Irregular time, Recurrence, Visit profiles, Longitudinal, Cancer

## Abstract

**Background:**

A variety of methods exist for the analysis of longitudinal data, many of which are characterized with the assumption of fixed visit time points for study individuals. This, however is not always a tenable assumption. Phenomenon that alter subject visit patterns such as adverse events due to investigative treatment administered, travel or any other emergencies may result in unbalanced data and varying individual visit time points. Visit times can be considered informative, because subsequent or current subject outcomes can change or be adapted due to previous subject outcomes.

**Methods:**

In this paper, a Bayesian Bernoulli-Exponential model for analyzing joint binary outcomes and exponentially distributed informative visit times is developed. Via statistical simulations, the influence of controlled variations in visit patterns, prior and sample size schemes on model performance is assessed. As an application example, the proposed model is applied to a Bladder Cancer Recurrence data.

**Results and conclusions:**

Results from the simulation analysis indicated that the Bayesian Bernoulli-Exponential joint model converged in stationarity, and performed relatively better for small to medium sample size scenarios with less varying time sequences regardless of the choice of prior. In larger samples, the model performed better for less varying time sequences. This model’s application to the bladder cancer data showed a statistically significant effect of prior tumor recurrence on the probability of subsequent recurrences.

## Introduction

Longitudinal data entail observations collected repeatedly on subjects over time. In medical research, the collection of correlated, longitudinal data is a common phenomenon. Ranging from the assessment of response changes and trends over time to understanding disease progression, the benefits longitudinal approaches are enormous [1, 2]. A defining feature of longitudinal data is the dependency that characterizes observations extending over time, the type of outcome measured and sometimes, the assumption of fixed time measurements for subjects [3–5]. The broad assumption of fixed time measurements, predetermined by study design, however is not always a tenable assumption. For instance, in a clinical trial, there is the potential for different visit mechanisms. Study subjects are likely to miss scheduled visits, and a proportion of them are prone to adverse events from investigative treatments. Also, due to poor health conditions, individuals may self elect to visit the investigative site or hospital more intensely than their study counterparts. These occurrences may result not just in unbalanced data for subjects, but also varying visit profiles. Thus, the time structure adopted for the study can be considered informative. In a broad sense, this indicates that outcomes measured at subsequent time points are influenced or can be adapted based on outcomes measured in current time. This necessitates the use of advanced methods that address the informative time structure rather than standard, traditional approaches, which are limited by the assumption of fixed time. To handle such scenarios, Bronsert [6] developed a classical joint model, involving Gaussian outcomes and exponentially distributed informative time. Later, Alomair [7] extended Bronsert’s model to include time dependent covariates. Classical informative time joint models have also been developed by Seo [8], involving longitudinal outcomes from the exponential families and exponentially distributed informative time. These joint models used the maximum likelihood estimation approach for estimating model parameters, and the authors broadly discussed associated computational complexities.

A Bayesian technique for modeling joint longitudinal outcomes and informative time points has been developed by Zaagan [9] but only for Gaussian distributed outcomes. The objectives of this research paper are twofold. First, we develop a Bayesian joint model for analyzing binary longitudinal outcomes and informative times. Then, via statistical simulations, we examine the influence of controlled variations in subject visit patterns, different prior specifications and sample size schemes on the proposed model. This proceeds with model convergence assessment and model evaluation. The proposed Bayesian-Exponential joint model is applied to a Bladder cancer recurrence data resulting from a clinical trial involving patients with bladder cancer conducted by the Veterans Administration Co-operative Urological Research Group (VACURG) [10, 11].

## Data and methods

### The Bayesian Bernoulli-Exponential joint model formulation and likelihood specification

The exponential family of distributions covers a broad range of response distributions including Gaussian and Non-Gaussian distributions [12, 13]. For example, the Normal, Gamma, Poisson, Bernoulli, and Beta distributions are a part of the parametric set of distributions included in the family. Suppose the observations $y_{1},y_{2},y_{3},\cdots , y_{n}$ are independent observations of a response variable, the exponential family of distributions from which the independent observations are sampled, can be specified as

1 $$\begin{aligned} f\left( y_{i} \mid \theta _{i}, \phi \right) =\exp \left\{ m_{i}^{-1}(\phi )\left( y_{i} \theta _{i}-s\left( \theta _{i}\right) \right) +r\left( y_{i}, \phi \right) \right\} ~~~i=1,\ldots , n. \end{aligned}$$

Where,

- $\theta _{i}$ represents the canonical parameter.
- $\phi$ is a scale parameter and $m_{i}(\cdot ), s(\cdot )$, and $r(\cdot )$ are known functions which relates to the variances of distributions in the exponential family.
- $m_{i}(\phi )$ can be specified as $m_{i}(\phi )=\frac{\phi }{u_{i}}$, and $u_{i}$ ’s are predetermined weights.

The canonical or location parameter characterizes a so called canonical link function, and relates to the means of the distributions in the exponential family.

Assume we have a set of *n* participants enrolled in a clinical trial, have to visit an investigative site over time and are followed over an interval from $(0,\tau ]$. A response observation for the *i*th participant measured at the *k*th visit time point can be specified as $y_{ik}$. We can further specify vectors of individual responses and their associated visit time points as

$$\begin{aligned} \varvec{y_{i}} = \left( \begin{array}{c} y_{i 1}\\ y_{i2}\\ y_{i3}\\ \vdots \\ y_{i n_{i}} \end{array}\right) , \qquad \qquad \quad \varvec{t_{i}} = \left( \begin{array}{c} t_{i 1}\\ t_{i 2}\\ t_{i 3}\\ \vdots \\ t_{i n_{i}}\\ \end{array}\right) . \end{aligned}$$

Here, the subscript $n_{i}$ allows for varying participant visit times. We can thus specify the joint distribution of recorded responses and time points as

2 $$\begin{aligned} f_{\Theta }\left( \varvec{y}_{i} , \varvec{t}_{i}\right) =f_{\Theta }\left( \varvec{y}_{i} \mid \varvec{t}_{i}\right) \cdot f_{\Theta }\left( \varvec{t}_{i}\right) , \end{aligned}$$

where $\Theta$ is a vector of unknown parameters to be estimated. Using these ideas, and in line with Seo [8] we can further specify a model that incorporates the joint distribution of responses and time points $\varvec{y}_{ik}$ and $\varvec{t}_{i_{n}}$ with the underlying assumption that the current response depends on the one-step prior response $\left( y_{ik-1}\right)$, and current visit time point $\left( t_{i k}\right)$. It is important to note, however, that subsequent responses, $y_{i k}$ will not be solely conditioned on observation time, $t_{i k}$ but also on the most recent prior response, $y_{ik-1}$ and observation time. This distribution can be specified as;

3 $$\begin{aligned} f_{\Theta }\left( \varvec{y}_{i}, \varvec{t}_{i}\right) =f_{\Theta }\left( y_{i 1} \mid t_{i 1}\right) \times f_{\Theta }\left( t_{i 1}\right) \times \prod _{k=2}^{n_{i}} f_{\Theta }\left( y_{i k} \mid t_{ik},t_{ik-1}, y_{ik-1}\right) \times f_{\Theta }\left( t_{ik} \mid y_{i k-1}\right) . \end{aligned}$$

This formulation forms the premise for specifying the joint model with response observations sampled from the Bernoulli distribution. Time is considered informative and assumed to be exponentially distributed. The joint distribution for binary longitudinal outcomes and informative time given the underlying assumption of a one step dependency can be specified as;

4 $$\begin{aligned} f_{\Theta }\left( \varvec{y}_{i}, \varvec{t}_{i}\right) = f\left( y_{ik} , y_{i k-1}, t_{i k},t_{ik-1}, \varvec{X}\right)= & {} \mu _{i k}^{y_{i k}}\left( 1-\mu _{i k}\right) ^{1-y_{i k}} \nonumber \\= & {} \exp \left( y_{i k} \log \left( \frac{\mu _{i k}}{1-\mu _{i k}}\right) +\log \left( 1-\mu _{i k}\right) \right) . \end{aligned}$$

Note that, $$\mu _{ik}=E\left( Y_{ik}\right) =P\left( Y_{ik}=1\right)$$.

More specifically for the Bernoulli distribution the link function can be specified as a logit link

5 $$\begin{aligned} \theta _{ik}=\log \left( \frac{\mu _{ik}}{1-\mu _{i k}}\right) =\text {logit}\left( \mu _{ik}\right) . \end{aligned}$$

which in the context of this study can be expressed as;

6 $$\begin{aligned} \log \left( \frac{\mu _{i j}}{1-\mu _{ik}}\right) =\varvec{X}_i^{\prime } \varvec{\alpha }+\vartheta t_{ik}+\psi y_{ik-1} . \end{aligned}$$

Furthermore, the specified mean function for the initial value for the *i*th participant and that after the initial value can be expressed as

$$\begin{aligned} \mu _{i 1}=\frac{\exp \left( \varvec{X}_i^{\prime } \varvec{\alpha }\right) }{1+\exp \left( \varvec{X}_i^{\prime } \varvec{\alpha }\right) } \quad \text{ and } \quad \mu _{ik}=\frac{\exp \left( \varvec{X}_i^{\prime } \varvec{\alpha }+\vartheta t_{ik}+\psi y_{ik-1}\right) }{1+\exp \left( \varvec{X}_i^{\prime } \varvec{\alpha }+\vartheta t_{ik}+\psi y_{ik-1}\right) .} \end{aligned}$$

respectively. Hence, our final model specification for the parametric joint Bernoulli-Exponential model can be expressed as;

7 $$\begin{aligned} f_{\Theta }\left( \varvec{y}_{i}, \varvec{t}_{i}\right)&= f\left( y_{i k} \mid y_{ik-1}, t_{ik},t_{ik-1}, \varvec{X}\right) = \exp \left\{ y_{i1} \ln \left( \frac{\mu _{i1}}{1-\mu _{i1}}\right) +\ln (1-\mu _{i1})\right\} \nonumber \\&\quad \times \prod _{k=2}^{n_{i}}\Bigg \{\exp \left( y_{ik} \ln \left( \frac{\mu _{ik}}{1-\mu _{ik}}\right) +\ln (1-\mu _{ik})\right) \nonumber \\&\quad \times \exp \left( \xi +\gamma y_{ik-1}\right) \times \exp \left( -\exp \left( \xi +\gamma y_{ik-1}\right) t_{ik}\right) \Bigg \}, ~~~~~ t_{ik} \in (0, \tau ]. \end{aligned}$$

Where,

- $\varvec{\alpha }$ is a vector of regression parameters denoting the effect of covariates on observed responses.
- $\psi$ represents the effect of the prior responses on average current responses.
- $\vartheta$ represents the effect of current response time on the mean responses,
- $\xi$ is a constant parameter associated with time
- $\gamma$ characterizes the effect of previous response on mean time and $\varvec{X}$ is the design matrix.

The resulting likelihood function, a product of the density functions for *s* subjects, can be specified as,

8 $$\begin{aligned} L\big (\Theta , y_{1},y_{2},y_{3} \cdots , y_{s}\big )&=\prod _{i=1}^{s}\Bigg \{\exp \bigg \{y_{i1} \ln \left( \frac{\mu _{i1}}{1-\mu _{i1}}\right) +\ln (1-\mu _{i1})\bigg \}\nonumber \\&\quad \times \prod _{k=2}^{n_{i}} \exp \Big \{y_{ik} \ln \left( \frac{\mu _{ik}}{1-\mu _{ik}}\right) +\ln (1-\mu _{ik})\nonumber \\&\quad \times \exp \left( \xi +\gamma y_{ik-1}\right) \times \exp \left( -\exp \left( \xi +\gamma y_{ik-1}\right) t_{ik}\right) \Big \}\Bigg \}. \end{aligned}$$

It is further important to clarify, that one key underlying assumption of this model, following Lin and Ying [14],Lin, Scharfstein, and Rosenheck [15], Liang, Wenbin and Zhiliang [16] and Sun, Sun, and Liu [17], is that censoring time, $Z_i$ in this study is noninformative in the sense that given covariates $\left( X_i\right) , Z_i$ is independent of the observation times $\left\{ t_{i k}\right.$, $k \ge 1\}$ and longitudinal outcomes $Y_i(\cdot )$. This basically means that given the covariate history up to time *k*, the distribution of the future covariate path up to any time $t>k$ is independent of whether or not there is an observation on $X_i$ at time *k*.

### Specification of priors

After the likelihood function of the Bernoulli-Exponential joint model distribution has been specified, the next step in the Bayesian model specification is the identification of a suitable prior. In this study, informative and non-informative priors are considered. Both priors serve important roles in Bayesian analysis, and the choice between them depends on the specific goals and available information in a given analysis [18]. Non-informative priors, also known as weak,vague or diffuse priors, are designed to have minimal influence on the posterior distribution. They can make Bayesian analysis robust to situations where there is little prior information or when prior beliefs are uncertain. They prevent strong prior assumptions from biasing results when there is limited prior knowledge [19]. One of the primary benefits of informative priors, on the other hand, is that they allow to incorporate expert domain knowledge and prior information into the analysis [20, 21]. This is invaluable when experts have insights that can improve parameter estimation, and, in situations with limited or noisy data, informative priors can lead to more stable and accurate parameter estimates. Finally, informative priors explicitly quantify prior beliefs and uncertainty, which allows to integrate these beliefs with observed data. In this study, for both informative and non-informative prior scenarios, we consider the vector of mean parameters $(\varvec{\alpha })$ as having a multivariate normal distribution [19, 22–24]. This is specified as;

9 $$\begin{aligned} p(\varvec{\alpha } \mid \phi )&\sim N\left( \varvec{\mu }_{\alpha }, \phi \varvec{\Sigma }_{\alpha }\right) \nonumber \\ p(\varvec{\alpha }\mid \phi )&=\bigg (2 \pi \bigg )^{\frac{s}{2}}\bigg |\phi \varvec{\Sigma }_{\alpha }\bigg |^{-\frac{1}{2}} \exp \left( -\frac{1}{2}\left( \varvec{\alpha }-\varvec{\mu }_{\alpha }\right) ^{\prime }\left( \phi \varvec{\Sigma }_{\alpha }\right) ^{-1}\left( \varvec{\alpha }-\varvec{\mu }_{\alpha }\right) \right) . \end{aligned}$$

Furthermore, we consider the parameters associated with time or visit to similarly follow a Gaussian distribution;

$$\begin{aligned} \gamma&\sim N\left( \mu _{\gamma }, \nu _{\gamma }^{2}\right) \\ \vartheta&\sim N\left( \mu _{\vartheta }, \nu _{\vartheta }^{2}\right) \\ \psi&\sim N\left( \mu _{\psi }, \nu _{\psi }^{2}\right) \\ \xi&\sim N\left( \mu _{\xi }, \nu _{\xi }^{2}\right) .\\ \end{aligned}$$

Note that the prior distributions of our joint model parameters are considered independent and thus,

10 $$\begin{aligned} p(\varvec{\alpha }, \vartheta ,\psi ,\xi )&=p(\varvec{\alpha }|\phi )\times p(\vartheta )\times p(\psi )\times p(\xi )\nonumber \\&=\bigg (2 \pi \bigg )^{\frac{s}{2}}\bigg |\phi \varvec{\Sigma }_{\alpha }\bigg |^{-\frac{1}{2}} \exp \left( -\frac{1}{2}\left( \varvec{\alpha }-\varvec{\mu }_{\alpha }\right) ^{\prime }\left( \phi \varvec{\Sigma }_{\alpha }\right) ^{-1}\left( \varvec{\alpha }-\varvec{\mu }_{\alpha }\right) \right) \nonumber \\&\quad\times \frac{1}{\sqrt{2 \pi \nu _{\vartheta }^{2}}} \exp \left( -\frac{1}{2}\left( \vartheta -\mu _{\vartheta }\right) ^{2}\right) \times \frac{1}{\sqrt{2 \pi \nu _{\psi }^{2}}} \exp \left( -\frac{1}{2}\left( \psi -\mu _{\psi }\right) ^{2}\right) \nonumber \\&\quad\times \frac{1}{\sqrt{2 \pi \nu _{\xi }^{2}}} \exp \left( -\frac{1}{2}\left( \xi -\mu _{\xi }\right) ^{2}\right) . \end{aligned}$$

For the informative prior setting, fixed values for the prior means, $(\varvec{\mu }_{\alpha },{\mu }_{\vartheta },{\mu }_{\psi },{\mu }_{\xi },{\mu }_{\omega })$ and their corresponding variances $(\varvec{\Sigma }_{\alpha },\nu _{\vartheta },\nu _{\psi },\nu _{\xi },\nu _{\omega })$ are adopted, since we do not have expert or historical estimates yet for these kind of studies. More specifically, we can denote the mean vector of $\varvec{\alpha }$, $\varvec{\mu }_{\alpha }$ with a prior mean vector and corresponding covariance matrix as;

$$\begin{aligned} p(\varvec{\alpha } \mid \phi )\sim N\left( 0.6 \varvec{\text{I}}_{s}, 5\varvec{\text{I}}_{s}\right) . \end{aligned}$$

where $\text{I}_{s}$ represents an identity matrix whose dimension depends on *s* individuals and $\phi$. More broadly, we set predetermined prior mean values for the visit parameters as;

$$\begin{aligned} ({\mu }_{\vartheta },{\mu }_{\psi },{\mu }_{\xi },{\mu }_{\omega })=(0.2,0.3,0.3,1), \end{aligned}$$

and their corresponding prior variances as

$$\begin{aligned} ({\nu }_{\vartheta }^{2},{\nu }_{\psi }^{2},{\nu }_{\xi }^{2},{\nu }_{\omega }^{2})=(0.3,0.4,0.2,1.5). \end{aligned}$$

Regarding the non-informative prior setting, two approaches are considered. First, Gaussian non-informative priors are adopted for all mean and variance parameters of both the response and time parameters. More broadly, to express prior ignorance, the prior means $({\mu }_{\alpha },{\mu }_{\vartheta },{\mu }_{\psi },{\mu }_{\xi },{\mu }_{\omega })$ are set to zero and the variance-covariance for $\phi \varvec{\Sigma }_{\alpha }$ can be set as a diagonal matrix with large variance. Similarly the corresponding prior variances for the other parameters are set very large to express prior ignorance. Thus, the non-informative priors are set up as,

$$\begin{aligned} p(\varvec{\alpha })&\sim N\left( \varvec{\text{0}}_{s}, 10^{8}\varvec{\text{I}}_{s}\right) \\ p({\gamma })&\sim N\left( 0, 10^{8}\right) \\ p(\vartheta )&\sim N\left( 0, 10^{8}\right) \\ p(\psi )&\sim N\left( 0, 10^{8}\right) \\ p(\xi )&\sim N\left( 0, 10^{8}\right) \\ p(\omega )&\sim N\left( 0, 10^{8}\right) . \end{aligned}$$

For the second case of non-informative prior, we consider the Jeffreys prior [25] an appealing reference prior widely used in Bayesian inference. This prior is considered for the response/outcome parameters and Gaussian non-informative priors are still considered in this study for visit parameters. The Jeffreys prior is obtained by applying the Jeffreys rule which defines the prior density to be directly proportional to the square root of the determinant of the Fisher information matrix. That is, for a set of parameters $\varvec{\theta }=\left( \theta _{1}, \ldots , \theta _{n}\right)$, the Jeffreys prior is given by,

$$\begin{aligned} p(\varvec{\theta }) \propto \bigg ({\text {det}( I(\varvec{\theta }))}\bigg )^{\frac{1}{2}}. \end{aligned}$$

The Fisher information matrix is defined by,

11 $$\begin{aligned} I(\varvec{\theta })=-E\left[ \frac{\partial ^{2} \ln L}{\partial \theta _{i} \partial \theta _{k}}\right] . \end{aligned}$$

and *L* is the likelihood function that specifies the probability for data *y* given the parameters $\theta$. It is appropriate so far as $\textbf{I}(\theta )$ is positive definite. Aside its geometric interpretation, one of the appealing reasons for its usage is the concept of parameterization invariance [26]. This means that the prior is invariant with regards to one-to-one transformations. The principle can be extended for multidimensional parameters. To establish ideas for the Jeffreys prior for response parameters, which result from the exponential family of distributions, the likelihood functions of the distributions and associated score vectors need to be specified.

Let $\phi _{i}$’s be known and $\varvec{X}^{\prime }$ assume a rank *q*. Also let, $\theta _{i}=z\left( \varvec{x}_{i}^{\prime } \varvec{\alpha }\right)$ and $m^{-1}\left( \phi _{i}\right) =\phi ^{-1}w$. The likelihood function for Generalized linear models with responses from the exponential family of distributions can generally be specified as;

12 $$\begin{aligned} L(\varvec{\alpha }) \propto \exp \left[ \sum _{i=1}^{n} m^{-1}\left( \phi _{i}\right) \left\{ y_{i} z\left( \varvec{x}_{i}^{\prime } \varvec{\alpha }\right) -s\left( z\left( \varvec{x}_{i}^{\prime } \varvec{\alpha }\right) \right) \right\} \right] . \end{aligned}$$

The score vector is represented by;

13 $$\begin{aligned} \frac{\partial \log L(\varvec{\alpha })}{\partial \varvec{\alpha }}=\sum _{i=1}^{n} m_{i}^{-1}\left( \phi _{i}\right) \left\{ y_{i}-s^{\prime }\left( z\left( \varvec{x}_{i}^{\prime } \varvec{\alpha }\right) \right) \right\} z^{\prime }\left( \varvec{x}_{i}^{\prime } \varvec{\alpha }\right) \varvec{x}_{i}. \end{aligned}$$

The resulting Fisher information matrix is specified as;

14 $$\begin{aligned} \varvec{I(\alpha )}=E\left[ -\frac{\partial ^{2} \log L}{\partial \varvec{\alpha } \partial \varvec{\alpha }^{\prime }}\right] =\varvec{X}^{\prime } \varvec{P} \varvec{V}(\varvec{\alpha }) \Delta ^{2}(\varvec{\alpha }) \varvec{X}. \end{aligned}$$

Here,

- $\varvec{P}=\text {Diag}\left( m^{-1}\left( \phi _{i}\right) ,\cdots , m^{-1}\left( \phi _{n}\right) \right)$ which is an $n \times n$ diagonal matrix of the weights $w_{i}$.
- $\varvec{V}(\varvec{\alpha })=\text {Diag}\left( s^{\prime \prime }\left( \left( \varvec{x}_{1}^{\prime } \varvec{\alpha }\right) \right) , \cdots , s^{\prime \prime }\left( \left( \varvec{x}_{n}^{\prime } \varvec{\alpha }\right) \right) \right)$ which reflects an $n \times n$ diagonal matrix of $v_{i}=$ $\frac{\partial ^{2} s\left( \theta _{i}\right) }{\partial \theta _{i}^{2}}$.
- $\varvec{\Delta }(\varvec{\alpha })=\text {Diag}\left( s^{\prime }\left( \varvec{x}_{1}^{T} \varvec{\alpha }\right) , \cdots , s^{\prime }\left( \varvec{x}_{n}^{T} \varvec{\alpha }\right) \right)$ is an a $n \times n$ diagonal matrix of $\delta _{i}=\frac{\partial s\left( \theta _{i}\right) }{\partial \eta _{i}}$ and is an adjustment for the link function.

The Jeffreys prior thus for $\varvec{\alpha }$ assuming $\phi$ is known, is specified as

15 $$\begin{aligned} p(\varvec{\alpha }) \propto \left| \varvec{X}^{\prime } \varvec{P} \varvec{V}(\varvec{\alpha }) \Delta ^{2}(\varvec{\alpha }) \varvec{X}\right| ^\frac{1}{2}. \end{aligned}$$

Based on this derivation, Jeffreys non-informative prior considered for response parameters and Gaussian non-informative priors maintained for the visit parameters can be specified as;

16 $$\begin{aligned} p(\varvec{\alpha }, \vartheta ,\psi ,\xi )&=p(\varvec{\alpha }|\phi )\times p(\vartheta )\times p(\psi )\times p(\xi )\times p(\omega )\nonumber \\&=\left| \varvec{X}^{\prime } \varvec{P} \varvec{V}(\varvec{\alpha }) \Delta ^{2}(\varvec{\alpha }) \varvec{X}\right| ^\frac{1}{2}\nonumber \\&\quad \times \frac{1}{\sqrt{2 \pi \nu _{\vartheta }^{2}}} \exp \left( -\frac{1}{2}\left( \vartheta -\mu _{\vartheta }\right) ^{2}\right) \times \frac{1}{\sqrt{2 \pi \nu _{\psi }^{2}}} \exp \left( -\frac{1}{2}\left( \psi -\mu _{\psi }\right) ^{2}\right) \nonumber \\&\quad \times \frac{1}{\sqrt{2 \pi \nu _{\xi }^{2}}} \exp \left( -\frac{1}{2}\left( \xi -\mu _{\xi }\right) ^{2}\right) . \end{aligned}$$

### Posterior distribution specification and Bayesian joint parameter estimation

The next step in the Bayesian model development is the specification of the posterior distribution, which has a directly proportional relationship with the model likelihood and the priors specified. For the scenario where Gaussian priors are considered for both the response and visit parameters and also for both informative and non informative settings, the resulting Bayesian Bernoulli-Exponential joint model posterior specification can be obtained as;

17 $$\begin{aligned}{}&p(\varvec{\alpha }, \vartheta ,\psi ,\xi ,\phi |\varvec{Y}_{i},\varvec{t}_{i},\varvec{X} )=L\left( \Theta , y_{1},y_{2},y_{3} \cdots , y_{s}\right) \times p(\varvec{\alpha }|\phi )\times p(\vartheta )\times p(\psi )\times p(\xi )\nonumber \\&=\prod _{i=1}^{s}\Bigg \{\exp \bigg \{y_{i1} \ln \left( \frac{\mu _{i1}}{1-\mu _{i1}}\right) +\ln (1-\mu _{i1})\bigg \}\times \prod _{k=2}^{n_{i}} \exp \Big \{y_{ik} \ln \left( \frac{\mu _{ik}}{1-\mu _{ik}}\right) \nonumber \\&\quad +\ln (1-\mu _{ik})\times \exp \left( \xi +\gamma y_{ik-1}\right) \times \exp \left( -\exp \left( \xi +\gamma y_{ik-1}\right) t_{ik}\right) \Big \}\Bigg \}\nonumber \\&\quad \times \bigg (2 \pi \bigg )^{\frac{s}{2}}\bigg |\phi \varvec{\Sigma }_{\alpha }\bigg |^{-\frac{1}{2}} \exp \left( -\frac{1}{2}\left( \varvec{\alpha }-\varvec{\mu }_{\alpha }\right) ^{\prime }\left( \phi \varvec{\Sigma }_{\alpha }\right) ^{-1}\left( \varvec{\alpha }-\varvec{\mu }_{\alpha }\right) \right) \times \frac{1}{\sqrt{2 \pi \nu _{\vartheta }^{2}}} \exp \left( -\frac{1}{2}\left( \vartheta -\mu _{\vartheta }\right) ^{2}\right) \nonumber \\&\quad \times \frac{1}{\sqrt{2 \pi \nu _{\psi }^{2}}} \exp \left( -\frac{1}{2}\left( \psi -\mu _{\psi }\right) ^{2}\right) \times \frac{1}{\sqrt{2 \pi \nu _{\xi }^{2}}} \exp \left( -\frac{1}{2}\left( \xi -\mu _{\xi }\right) ^{2}\right) . \end{aligned}$$

Also for the scenario where Jeffreys priors are considered for the parameters of the Bernoulli response and Gaussian priors for the visit parameters (non informative settings), the resulting Bayesian Bernoulli-Exponential joint model can be parameterized as;

18 $$\begin{aligned} p(\varvec{\alpha }, \vartheta ,\psi ,\xi ,\phi |\varvec{Y}_{i},\varvec{t}_{i},\varvec{X} )=L\left( \Theta , y_{1},y_{2},y_{3} \cdots , y_{s}\right) \times p(\varvec{\alpha }|\phi )\times p(\vartheta )\times p(\psi )\times p(\xi ). \end{aligned}$$

$$\begin{aligned}{}&=\prod _{i=1}^{s}\Bigg \{\exp \bigg \{y_{i1} \ln \left( \frac{\mu _{i1}}{1-\mu _{i1}}\right) +\ln (1-\mu _{i1})\bigg \}\times \prod _{k=2}^{n_{i}} \exp \Big \{y_{ik} \ln \left( \frac{\mu _{ik}}{1-\mu _{ik}}\right) \\&\quad +\ln (1-\mu _{ik})\times \exp \left( \xi +\gamma y_{ik-1}\right) \times \exp \left( -\exp \left( \xi +\gamma y_{ik-1}\right) t_{ik}\right) \Big \}\Bigg \}.\\&\quad \times \left| \varvec{X}^{\prime } \varvec{P} \varvec{V}(\varvec{\alpha }) \Delta ^{2}(\varvec{\alpha }) \varvec{X}\right| ^\frac{1}{2}\times \frac{1}{\sqrt{2 \pi \nu _{\vartheta }^{2}}} \exp \left( -\frac{1}{2}\left( \vartheta -\mu _{\vartheta }\right) ^{2}\right) \\&\quad \times \frac{1}{\sqrt{2 \pi \nu _{\psi }^{2}}} \exp \left( -\frac{1}{2}\left( \psi -\mu _{\psi }\right) ^{2}\right) \times \frac{1}{\sqrt{2 \pi \nu _{\xi }^{2}}} \exp \left( -\frac{1}{2}\left( \xi -\mu _{\xi }\right) ^{2}\right) . \end{aligned}$$

Here, $\varvec{V}(\varvec{\alpha })=\text {diag}\left( v_{1},v_{2} \ldots , v_{n}\right)$ and $v_{i}=\mu _{ik}(1-\mu _{ik})$. Note that,

$$\begin{aligned} \mu _{ik}=\frac{\exp \left( \varvec{\alpha }^{\prime }\varvec{X}_{i} + \vartheta t_{ik}+\psi y_{i k-1}\right) }{1+\exp \left( \varvec{\alpha }^{\prime }\varvec{X}_{i} + \vartheta t_{ik}+\psi y_{i k-1}\right) }. \end{aligned}$$

The next goal is to obtain posterior summary estimates for inference. Analytical calculations of the posterior distributions are possible, but often untenable due to laborious calculations involving the integration constant. Integral approximation methods can be adopted but only if few parameters are involved [19, 24]. In situations such as this study involving many parameters to be estimated, one can resort to Markov Chain Monte Carlo Methods (MCMC) [27]. The MCMC methods are viable simulation approaches for sampling from posterior distributions and computing posterior summary measures. They are premised on a Markov Chain construction that subsequently converges to a so-called target distribution. The two most popular MCMC methods are the Gibbs sampling and the Metropolis-Hastings algorithm [27–29]. In this study, we adopt the Gibbs sampling procedure for generating samples from the joint posterior distributions of the unknown parameters in our model. It is important to clarify, however, that the Gibbs sampler, performs iterative draws from posterior conditional distributions instead of directly sampling from the joint posterior distribution. This approach enhances the utility of the Gibbs Sampler, especially when dealing with complex joint posteriors that can be challenging to handle directly. Then, posterior summaries can be computed. In each step of the algorithm, random values are generated from unidimensional distributions [30]. A brief summary of the Gibbs sampling algorithm is as follows;

- Predetermined initial values $\varvec{\theta }^{(0)}$ need to be specified.
- For $$t=1, \ldots , T$$ iterations,
  (i) Set $$\varvec{\theta }=\varvec{\theta }^{(t-1)}$$.
  (ii) For $k=1, \ldots , r$, we can update $\theta _{k}$ from $\theta _{k} \sim p\left( \theta _{k} \mid \theta _{1}, \ldots , \theta _{k-1}, \theta _{k+1}, \ldots , \theta _{r}\right)$.

Now, if the current state of the chain $\theta$ is $\theta ^{(t)}=\left( \theta _{1}^{(t)} \ldots , \theta _{r}\right)$, then we can generate the new parameters by,

$$\begin{aligned} \begin{array}{ccc} \text {Drawing}\ \ \theta _{1}^{(t)} &{} \text {from} &{} p\left( \theta _{1} \mid \theta _{2}^{(t-1)}, \theta _{3}^{(t-1)}, \ldots , \theta _{q}^{(t-1)}, \varvec{y}\right) , \\ \text {Drawing}\ \ \theta _{2}^{(t)} &{} \text {from} &{} p\left( \theta _{2} \mid \theta _{1}^{(t)}, \theta _{3}^{(t-1)}, \ldots , \theta _{q}^{(t-1)}, \varvec{y}\right) , \\ \text {Drawing}\ \ \theta _{3}^{(t)} &{} \text {from} &{} p\left( \theta _{3} \mid \theta _{1}^{(t)}, \theta _{2}^{(t)}, \theta _{4}^{(t-1)}, \ldots , \theta _{q}^{(t-1)}, \varvec{y}\right) ,\\ {} &{} \vdots &{} {}\\ \text {Drawing}\qquad \theta _{q}^{(t)} &{} \text {from} &{} p\left( \theta _{q} \mid \theta _{1}^{(t)}, \theta _{2}^{(t)}, \ldots , \theta _{q-1}^{(t)}, \varvec{y}\right) . \end{array} \end{aligned}$$

The distributions, $p\left( \theta _{k} \mid \theta _{1}^{(t)}, \theta _{2}^{(t)}, \ldots , \theta _{k-1}^{(t)}, \theta _{k+1}^{(t-1)}, \ldots , \theta _{q}^{(t-1)}, \varvec{y}\right)$ are known as the full, complete or conditional distributions. Summarily, the Gibbs sampling algorithm helps to iteratively generate samples from our posterior distribution based on prespecified starting values. Initial portions of the Markov chains are discarded in an attempt to mask the influence of initial values. This is called the burn-in part. Resulting posterior summary measures such as the posterior mean, posterior standard deviation and Bayesian credible intervals are obtained from the MCMC output. Furthermore, we assess convergence of the Markov chains via the diagnosis of ergodic mean plots of estimated parameters and the Heidelberger and Welch diagnostic test which is a more formal convergence diagnostic method [31].

### Model evaluation

To assess the Bayesian Bernoulli-Exponential joint model, the Bayesian model evaluation criteria called the Deviance Information Criterion (DIC) is used [32]. The DIC measure comprises a “goodness of fit” and “complexity” term and is obtained as;

$$\begin{aligned} \text{DIC}&=-2 \ln L [\varvec{y} \mid \text{E}(\varvec{\theta } \mid \varvec{y})]+2 p_{\text{D}},\\&=\hat{D}(\varvec{\theta })+2 p_{\text{D}}. \end{aligned}$$

where $\hat{D}(\varvec{\theta })$ is the deviance calculated at the posterior mean of the parameters and $p_{\text{D}}$ characterizes the “effective” number of parameters relating the complexity of the models. $p_{\text{D}}$ is the difference between the posterior mean deviance, $\overline{D(\varvec{\theta })}$ and deviance calculated at the posterior mean of the parameters, $\hat{D}(\varvec{\theta })$. Smaller values of DIC justify a better fit of the model. In line with this derivation, the DIC measure for the Bayesian Bernoulli-Exponential model is specified as;

$$\begin{aligned} \text {DIC}&=-2\Bigg \{\sum _{i=1}^{s} y_{i 1} \left( \varvec{\hat{\alpha }}^{\prime }\varvec{X}_{i}\right) +\ln \left( \frac{1}{1+\exp (\hat{\varvec{\alpha }}^{\prime }\varvec{X}_{i})}\right) \\&\quad +\sum _{i=1}^{s}\sum _{k=2}^{n_{i}} \left( y_{ik} \left( \varvec{\hat{\alpha }}^{\prime }\varvec{X}_{i} + \hat{\vartheta } t_{ik}+\hat{\psi } y_{i k-1}\right) +\ln \left( \frac{1}{1+\left( \varvec{\hat{\alpha }}^{\prime }\varvec{X}_{i} + \hat{\vartheta } t_{ik}+\hat{\psi } y_{i k-1}\right) }\right) \right) \\&\quad +\sum _{i=1}^{s}\sum _{k=2}^{n_{i}} \bigg ((\hat{\xi }+\hat{\gamma } y_{ik-1})-\exp \left( \hat{\xi }+\hat{\gamma } y_{ik-1}\right) t_{ik}\bigg )\Bigg \} +2 p_{\text{D}}. \end{aligned}$$

## Results

### Simulation study

In order to assess the Bayesian Bernoulli-Exponential model in terms of how it can be influenced by controlled variations in sample size, visit schema and types of prior distributions on the parameter estimates we present in this subsection, a simulation study. More precisely, the simulation study helps establish the validity of the joint model in random scenarios via data generation and parameter estimation. It is important to clarify, however, that this present study is an extension of the studies of Bronsert [6], Lin [33], Seo [8] and Zaagan [9] and thus for computational convenience, an abundant level of consistency is maintained in terms of simulation conditions. All simulations are performed in R software via the R2OpenBugs package. This package provides a means to program Bayesian models in R via an OpenBugs software [34, 35]. To develop the Bayesian joint model, the structure of the data to be simulated is clearly defined. We simulate data involving two categorical variables, each having three levels, and two continuous variables. The longitudinal responses are simulated from a Bernoulli distribution. The first response is simulated from the distribution, and then the subsequent response is computed based on the relationship between the prior outcome and the prior time for predicting the average response based on starting parameter values in Table 1. It is important to clarify, however that during the simulation exercise, only “plausible” starting values from the range of starting values in Table 1 are utilized. It is not the intent of this study to analyze the impact of all four range of starting values. The visit times for each of the corresponding responses are simulated from an exponential distribution.

**Table 1:** Parameter initial value scheme for simulations

$\varvec{\alpha}_{\varvec{1}}$	$\varvec{\alpha}_{\varvec{2}}$	$\varvec{\alpha}_{\varvec{3}}$	$\varvec{\alpha}_{\varvec{4}}$	$\varvec{\alpha}_{\varvec{5}}$	$\varvec{\alpha}_{\varvec{6}}$	$\varvec{\alpha}_{\varvec{7}}$	$\varvec{\psi}$	$\varvec{\vartheta}$	$\varvec{\xi}$	$\varvec{\gamma}$
0.4	0.2	0.3	0.1	0.3	0.4	0.9	0.8	0.1	2	0.01
0.4	0.2	0.3	0.1	0.3	0.4	0.9	0.8	0.1	1	0.02
0.4	0.2	0.3	0.1	0.3	0.4	0.9	0.8	0.1	2	0.01
0.4	0.2	0.3	0.1	0.3	0.4	0.9	0	0.1	1	0.02

Furthermore, we simulate design structures that consider varying visit schemes and sample sizes to effectively study trends or patterns associated with the model. In this study, three varying sample sizes with four sub design visit structures entailing both balanced and unbalanced visit structures are considered and shown in Table 2. Also, three prior schemes are considered, that is Gaussian informative, Gaussian non-informative and Jeffreys non-informative priors.

**Table 2:** Simulation design scheme

Scheme	Sample Size	Observations	Design Structure	Observation Totals
1	18	10	Balanced	180
2		5 & 3	Unbalanced	72
3		20 & 6	Unbalanced	234
4	54	10	Balanced	540
5		5 & 3	Unbalanced	216
6		20 & 6	Unbalanced	702
7	180	10	Balanced	1800
8		5 & 3	Unbalanced	720
9		20 & 6	Unbalanced	2340

Thus, the simulation matrix involves three varying sample size designs, three varying prior schemes and three visit design structures. To further clarify the visit structure, as an example to signal an unbalanced visit pattern, when the sample size is 180 and the number of observations is 20 & 6 , this exemplifies 90 participants having 20 recorded observations and another 90 subjects have 6 measured outcomes each. This simulation design scheme results in 27 differing designs for the simulation analysis of the Bayesian Bernoulli-Exponential joint model.

After data generation, the simulation analysis involves estimating the joint model parameters via the package R2Openbugs in R software. It commences by first “sinking” in generated parameter values which that serve as initial values for the MCMC estimation process. Then, the likelihood of the Bayesian joint model is calculated based on the design structures and priors specified. Parameter estimation proceeds with the Gibbs Sampling approach, which has earlier been discussed. This generates dependent Markov chains for our model parameters by drawing samples from the posterior distribution using initial parameter values that were embedded in the simulation design. Markov chains are run iteratively 30,000 times, and the first 10,000 iterations are discarded to serve as burn-in, effectively mitigating the influence of the initial values. Thinning intervals of three iterations are considered to monitor autocorrelations of the generated values. Subsequently, to monitor convergence of Markov chains and their associated posterior parameters, the Heidelberger and Welch convergence tests are computed. Then, posterior summaries such as the mean, standard deviation, and credible interval limits are presented. It is instructive to note that the simulations were replicated a 1000 times and inferences were premised on the averaged estimates and associated credible intervals. Finally, inferences via comparisons for different specification of the prior distribution and their sample size and visit design schemes for the model are made along with Deviance Information Criterion measures.

### Simulation results: model convergence assessment of the Bayesian Bernoulli-Exponential joint model

To evaluate convergence of the Markov chains of the model parameters, a formal diagnostic test, called the Heidelberger and Welch test [31] is used. It is expected that after the burn-in period, the Gibbs Sampling algorithm produces samples from the posterior distribution that attains a stationary distribution. The Heidelberger and Welch test constitutes a stationary and half-width test and calculates a test statistic to accept or reject the null hypothesis that the Markov chains are from a stationary distribution. The half-width test is based on a computed $95\%$ confidence interval for the mean, using the chain that earlier passed the stationarity test. The resulting ratio of the interval half-width and the mean compared with a threshold ( $\varepsilon =0.1$) determines whether the half-width test is passed or not. More precisely, the test passes if the ratio between the half-width and the mean is lesser than $\varepsilon$. Selected convergence results based on the Heidelberger and Welch test are presented for the Bayesian Bernoulli-Exponential joint model across select scenarios and shown in the Tables 3, 4 and 5. These results cut across all prior scenarios (informative, non-informative, Jeffreys non-informative Prior), sample sizes (18, 54, 180) and visit patterns (10, balanced), (5 &3, Unbalanced), (20 & 6 ,Unbalanced). Inferring from the Heidelberger and Welch tests conducted across the broad range of scenarios selected, no issues were observed with the convergence of the MCMC chains for the Bayesian Bernoulli-Exponential Joint model. More precisely, the *p*-values resulting from the stationarity test for all estimated model parameters, regardless of prior, sample size or visit schemes were statistically insignificant. This suggests that the sampled values for parameters are from a stationary process. A further indication is that our model parameter estimation can be implemented with precision because MCMC chains are in a stationary distribution.

**Table 3:** Heidelberger and welch test for the Bayesian Bernoulli-Exponential model and for the Gaussian informative prior

Sample Size and Design Structure	Parameter	Stationarity Test	*P*-value	Halfwidth Test	Halfwidth
18(10)	$\alpha _{1}$	passed	0.5613	passed	0.0131
	$\alpha _{2}$	passed	0.2289	passed	0.0085
	$\alpha _{3}$	passed	0.0831	passed	0.0082
	$\alpha _{4}$	passed	0.0821	passed	0.0083
	$\alpha _{5}$	passed	0.7699	passed	0.0083
	$\alpha _{6}$	passed	0.0632	passed	0.0075
	$\alpha _{7}$	passed	0.4511	passed	0.0052
	$\gamma$	passed	0.2434	passed	0.0135
	$\psi$	passed	0.0944	passed	0.0138
	$\vartheta$	passed	0.7248	passed	0.0024
	$\xi$	passed	0.2900	passed	0.0122
54(5 &3)	$\alpha _{1}$	passed	0.3399	passed	0.0107
	$\alpha _{2}$	passed	0.1035	passed	0.0077
	$\alpha _{3}$	passed	0.0690	passed	0.0079
	$\alpha _{4}$	passed	0.5958	passed	0.0078
	$\alpha _{5}$	passed	0.9991	passed	0.0086
	$\alpha _{6}$	passed	0.0837	passed	0.0038
	$\alpha _{7}$	passed	0.9054	passed	0.0033
	$\gamma$	passed	0.3158	passed	0.0094
	$\psi$	passed	0.2489	passed	0.0114
	$\vartheta$	passed	0.5081	passed	0.0025
	$\xi$	passed	0.4021	passed	0.0080
180(20 &6)	$\alpha _{1}$	passed	0.8574	passed	0.0093
	$\alpha _{2}$	passed	0.0874	passed	0.0047
	$\alpha _{3}$	passed	0.6287	passed	0.0048
	$\alpha _{4}$	passed	0.7431	passed	0.0047
	$\alpha _{5}$	passed	0.5685	passed	0.0051
	$\alpha _{6}$	passed	0.2030	passed	0.0014
	$\alpha _{7}$	passed	0.4465	passed	0.0017
	$\gamma$	passed	0.3473	passed	0.0032
	$\psi$	passed	0.4006	passed	0.0045
	$\vartheta$	passed	0.6792	passed	0.0008
	$\xi$	passed	0.3890	passed	0.0028

**Table 4:** Heidelberger and welch test for the bayesian bernoulli-exponential model and for the gaussian non-informative prior

Sample Size and Design Structure	Parameter	Stationarity Test	*P*-value	Halfwidth Test	Halfwidth
18(5 &3)	$\alpha _{1}$	passed	0.4006	passed	0.1123
	$\alpha _{2}$	passed	0.0614	passed	0.0455
	$\alpha _{3}$	passed	0.4879	passed	0.0637
	$\alpha _{4}$	passed	0.3864	passed	0.0608
	$\alpha _{5}$	passed	0.4574	passed	0.0581
	$\alpha _{6}$	passed	0.5380	passed	0.0352
	$\alpha _{7}$	passed	0.5282	passed	0.0186
	$\gamma$	passed	0.7625	passed	0.0174
	$\psi$	passed	0.0907	passed	0.0293
	$\vartheta$	passed	0.1241	passed	0.0088
	$\xi$	passed	0.7724	passed	0.0145
54(10)	$\alpha _{1}$	passed	0.0848	passed	0.0273
	$\alpha _{2}$	passed	0.1003	passed	0.0143
	$\alpha _{3}$	passed	0.1271	passed	0.0161
	$\alpha _{4}$	passed	0.1238	passed	0.0154
	$\alpha _{5}$	passed	0.0608	passed	0.0166
	$\alpha _{6}$	passed	0.1927	passed	0.0046
	$\alpha _{7}$	passed	0.0774	passed	0.0036
	$\gamma$	passed	0.4427	passed	0.0072
	$\psi$	passed	0.4846	passed	0.0108
	$\vartheta$	passed	0.9191	passed	0.0017
	$\xi$	passed	0.5784	passed	0.0068
180(20 &6)	$\alpha _{1}$	passed	0.6816	passed	0.0126
	$\alpha _{2}$	passed	0.1511	passed	0.0059
	$\alpha _{3}$	passed	0.3521	passed	0.0057
	$\alpha _{4}$	passed	0.7916	passed	0.0061
	$\alpha _{5}$	passed	0.3541	passed	0.0065
	$\alpha _{6}$	passed	0.2298	passed	0.0012
	$\alpha _{7}$	passed	0.6382	passed	0.0019
	$\gamma$	passed	0.2806	passed	0.0035
	$\psi$	passed	0.0817	passed	0.0055
	$\vartheta$	passed	0.1450	passed	0.0008
	$\xi$	passed	0.3527	passed	0.0031

**Table 5:** Heidelberger and welch test for the Bayesian Bernoulli-Exponential model and for the Jeffreys non-informative prior

Sample Size and Design Structure	Parameter	Stationarity Test	*P*-value	Halfwidth Test	Halfwidth
18(20 &6)	$\alpha _{1}$	passed	0.1046	passed	0.0473
	$\alpha _{2}$	passed	0.0901	passed	0.0231
	$\alpha _{3}$	passed	0.0878	passed	0.0273
	$\alpha _{4}$	passed	0.0555	passed	0.0273
	$\alpha _{5}$	passed	0.3411	passed	0.0238
	$\alpha _{6}$	passed	0.0693	passed	0.0197
	$\alpha _{7}$	passed	0.3220	passed	0.0096
	$\gamma$	passed	0.4874	passed	0.0120
	$\psi$	passed	0.3489	passed	0.0179
	$\vartheta$	passed	0.2100	passed	0.0030
	$\xi$	passed	0.4830	passed	0.0111
54(10)	$\alpha _{1}$	passed	0.0582	passed	0.0225
	$\alpha _{2}$	passed	0.3334	passed	0.0129
	$\alpha _{3}$	passed	0.1332	passed	0.0133
	$\alpha _{4}$	passed	0.0617	passed	0.0133
	$\alpha _{5}$	passed	0.1518	passed	0.0165
	$\alpha _{6}$	passed	0.0552	passed	0.0041
	$\alpha _{7}$	passed	0.0681	passed	0.0030
	$\gamma$	passed	0.2791	passed	0.0075
	$\psi$	passed	0.3487	passed	0.0096
	$\vartheta$	passed	0.8298	passed	0.0015
	$\xi$	passed	0.3672	passed	0.0068
180 (5 &3)	$\alpha _{1}$	passed	0.4216	passed	0.0115
	$\alpha _{2}$	passed	0.1339	passed	0.0053
	$\alpha _{3}$	passed	0.0623	passed	0.0055
	$\alpha _{4}$	passed	0.6780	passed	0.0060
	$\alpha _{5}$	passed	0.5003	passed	0.0063
	$\alpha _{6}$	passed	0.5941	passed	0.0016
	$\alpha _{7}$	passed	0.1024	passed	0.0016
	$\gamma$	passed	0.6136	passed	0.0032
	$\psi$	passed	0.0126	passed	0.0120
	$\vartheta$	passed	0.2572	passed	0.0009
	$\xi$	passed	0.5216	passed	0.0029

### Simulation results: parameter estimation and evaluation of the Bayesian Bernoulli-Exponential model

In this section, the influence of controlled variations in sample size, visit sequences and type of prior distributions on the estimated parameters of the Bayesian Bernoulli-Exponential model are examined. Consistency in the direction of these estimates and their associated credible intervals are checked. For ease of reporting, we present a select number of results from the various simulation scenarios. Posterior means, standard deviations and credible intervals of select scenarios are presented in Tables 6, 7, 8, 9 and 10.

**Table 6:** Table of parameter estimates for the Bayesian Bernoulli-Exponential joint model and for the Gaussian informative prior scheme

Sample Size and Design Structure	Table of Parameter Estimates				
	Parameter	Mean	SD	Lower(2.50%)	Upper(97.50%)
18(10)	$\alpha _{1}$	0.0612	0.3124	-0.6635	0.5512
	$\alpha _{2}$	0.3842	0.3373	-0.2775	1.0430
	$\alpha _{3}$	0.2442	0.3164	-0.3719	0.8610
	$\alpha _{4}$	0.5997	0.3387	-0.0470	1.2690
	$\alpha _{5}$	0.5423	0.3214	-0.0880	1.1660
	$\alpha _{6}$	0.4866	0.2678	-0.0207	1.0200
	$\alpha _{7}$	0.6965	0.2201	0.2654	1.1330
	$\gamma$	-1.1970	0.4424	-2.0670	0.3595
	$\psi$	0.0821	0.1880	-0.2705	0.4692
	$\vartheta$	-0.0424	0.0945	-0.2403	0.1355
	$\xi$	-1.0138	0.1675	-1.3610	-0.7008
54(5 &3)	$\alpha _{1}$	-0.1757	0.2720	-0.7142	0.3419
	$\alpha _{2}$	0.1808	0.2991	-0.4001	0.7733
	$\alpha _{3}$	0.3036	0.2979	-0.2831	0.8919
	$\alpha _{4}$	0.1360	0.3060	-0.4670	0.7350
	$\alpha _{5}$	0.2789	0.3015	-0.3050	0.8738
	$\alpha _{6}$	0.3109	0.1828	-0.0454	0.6655
	$\alpha _{7}$	0.8236	0.1811	0.4837	1.1830
	$\gamma$	-0.1481	0.1699	-0.4760	0.1882
	$\psi$	-0.3989	0.3829	-1.1540	0.3522
	$\vartheta$	-0.1782	0.0481	-0.2800	-0.0907
	$\xi$	-2.0490	0.1452	-2.3330	-1.7800
180(20 &6)	$\alpha _{1}$	0.8981	0.5843	-0.1935	2.0410
	$\alpha _{2}$	-0.1049	0.6234	-1.3520	1.0780
	$\alpha _{3}$	-0.4832	0.5375	-1.5330	0.5700
	$\alpha _{4}$	-0.4959	0.5042	-1.4250	0.4862
	$\alpha _{5}$	-0.0917	0.5033	-1.0750	0.8553
	$\alpha _{6}$	-0.0368	0.3831	-0.7801	0.7299
	$\alpha _{7}$	1.0100	0.3290	0.4111	1.6750
	$\gamma$	-0.2809	0.1697	-0.6027	0.0597
	$\psi$	-1.3460	0.3874	-2.0940	-0.6185
	$\vartheta$	-0.1198	0.0926	-0.3091	0.0491
	$\xi$	-0.6537	0.1535	-0.9614	-0.3621

**Table 7:** Table of parameter estimates for the Bayesian Bernoulli-Exponential joint model and for the Gaussian non-informative prior scheme

Sample Size and Design Structure	Table of Parameter Estimates				
	Parameter	Mean	SD	Lower$\varvec{(2.50\%)}$	Upper$\varvec{(97.50\%)}$
18(5 &3)	$\alpha _{1}$	1.0670	1.0670	-1.8300	2.4030
	$\alpha _{2}$	1.0380	1.0380	-1.9600	2.0430
	$\alpha _{3}$	0.9729	0.9729	-1.8480	2.0490
	$\alpha _{4}$	1.0700	1.0700	-2.5220	1.6180
	$\alpha _{5}$	0.9262	0.9262	-3.1460	0.5070
	$\alpha _{6}$	0.7034	0.7034	-0.4158	2.3020
	$\alpha _{7}$	0.5457	0.5457	-0.0391	2.0600
	$\gamma$	0.3015	0.3015	-0.5464	0.6282
	$\psi$	0.8522	0.8522	-3.4690	-0.0715
	$\vartheta$	0.0763	0.0763	-0.2946	0.0040
	$\xi$	0.2487	0.2487	-2.6140	-1.6530
54(10)	$\alpha _{1}$	0.4482	0.3309	-0.2180	1.0450
	$\alpha _{2}$	-0.1806	0.2985	-0.7503	0.3909
	$\alpha _{3}$	0.0118	0.3006	-0.5744	0.5926
	$\alpha _{4}$	0.1175	0.2906	-0.4452	0.6903
	$\alpha _{5}$	0.4809	0.3315	-0.1621	1.1480
	$\alpha _{6}$	0.0819	0.1293	-0.1749	0.3317
	$\alpha _{7}$	0.9209	0.1375	0.6562	1.1900
	$\gamma$	-0.0814	0.1127	-0.2949	0.1383
	$\psi$	-0.6282	0.2572	-1.1400	-0.1231
	$\vartheta$	-0.2123	0.0614	-0.3351	-0.0954
	$\xi$	-0.9536	0.1007	-1.1520	-0.7671
180(20 &6)	$\alpha _{1}$	0.8981	0.5843	-0.1935	2.0410
	$\alpha _{2}$	-0.1049	0.6234	-1.3520	1.0780
	$\alpha _{3}$	-0.4832	0.5375	-1.5330	0.5700
	$\alpha _{4}$	-0.4959	0.5042	-1.4250	0.4862
	$\alpha _{5}$	-0.0917	0.5033	-1.0750	0.8553
	$\alpha _{6}$	-0.0368	0.3831	-0.7801	0.7299
	$\alpha _{7}$	1.0100	0.3290	0.4111	1.6750
	$\gamma$	-0.2809	0.1697	-0.6027	0.0597
	$\psi$	-1.3460	0.3874	-2.0940	-0.6185
	$\vartheta$	-0.1198	0.0926	-0.3091	0.0491
	$\xi$	-0.6537	0.1535	-0.9614	-0.3621

**Table 8:** Table of parameter estimates for the Bayesian Bernoulli-Exponential joint model and for the Jeffreys non-informative prior scheme

Sample Size and Design Structure	Table of Parameter Estimates				
	Parameter	Mean	SD	Lower$\varvec{(2.50\%)}$	Upper$\varvec{(97.50\%)}$
18(20 &6)	$\alpha _{1}$	-0.3553	0.5372	-1.3870	0.6201
	$\alpha _{2}$	0.4144	0.5797	-0.7269	1.5560
	$\alpha _{3}$	0.0363	0.5138	-0.9527	1.0400
	$\alpha _{4}$	0.9047	0.5448	-0.1325	2.0140
	$\alpha _{5}$	0.7546	0.5100	-0.2522	1.7900
	$\alpha _{6}$	-0.2815	0.3920	-1.0060	0.5173
	$\alpha _{7}$	0.4952	0.2707	-0.0085	1.0270
	$\gamma$	-0.1025	0.1734	-0.4249	0.2390
	$\psi$	-1.7320	0.4147	-2.5520	-0.9451
	$\vartheta$	-0.2822	0.1176	-0.5226	-0.0653
	$\xi$	-0.8246	0.1587	-1.1450	-0.5167
54(10)	$\alpha _{1}$	0.1051	0.2939	-0.4522	0.6736
	$\alpha _{2}$	-0.1363	0.2784	-0.6854	0.4119
	$\alpha _{3}$	0.2195	0.2881	-0.3424	0.7763
	$\alpha _{4}$	0.1937	0.2873	-0.3593	0.7665
	$\alpha _{5}$	0.1010	0.2921	-0.4732	0.6801
	$\alpha _{6}$	0.4376	0.1256	0.1903	0.6834
	$\alpha _{7}$	0.5031	0.1121	0.2888	0.7236
	$\gamma$	-0.1627	0.1068	-0.3688	0.0468
	$\psi$	-1.1910	0.2371	-1.6420	-0.7255
	$\vartheta$	-0.0887	0.0470	-0.1835	0.0018
	$\xi$	-0.9061	0.0937	-1.0930	-0.7318
180(5 &3)	$\alpha _{1}$	-0.0121	0.2217	-0.4340	0.4203
	$\alpha _{2}$	-0.0042	0.2247	-0.4505	0.4319
	$\alpha _{3}$	0.3473	0.2343	-0.1066	0.8064
	$\alpha _{4}$	0.4331	0.2350	-0.0284	0.8947
	$\alpha _{5}$	0.3283	0.2293	-0.1188	0.7739
	$\alpha _{6}$	0.1849	0.0936	0.0023	0.3670
	$\alpha _{7}$	0.5730	0.1032	0.3746	0.7754
	$\gamma$	-0.0852	0.0941	-0.2701	0.0956
	$\psi$	-0.7435	0.2031	-1.1410	-0.3454
	$\vartheta$	-0.1052	0.0211	-0.1486	-0.0645
	$\xi$	-1.9430	0.0792	-2.0980	-1.7920

**Table 9:** Credible interval widths for selected scenarios for the Bernoulli-Exponential model

Prior Scenario	Confidence Interval Widths for Scenarios			
	Parameter	18(10)	54(10)	180(10)
Non-informative prior	$\alpha _{1}$	2.5230	1.2630	0.5893
	$\alpha _{2}$	2.5233	1.1412	0.5893
	$\alpha _{3}$	2.2581	1.1670	0.5789
	$\alpha _{4}$	2.2239	1.1355	0.5818
	$\alpha _{5}$	2.0500	1.3101	0.6033
	$\alpha _{6}$	1.6099	0.5066	0.2313
	$\alpha _{7}$	1.1259	0.5338	0.2711
	$\gamma$	0.7179	0.4332	0.2168
	$\psi$	1.6386	1.0169	0.9782
	$\vartheta$	0.3937	0.2397	0.1097
	$\xi$	0.6261	0.3849	0.1878

**Table 10:** Table of parameter estimates for the Bayesian Bernoulli-Exponential joint model and for the Jeffreys non-informative prior scheme

Sample Size and Design Structure	Table of Parameter Estimates				
	Parameter	Mean	SD	Lower$\varvec{(2.50\%)}$	Upper$\varvec{(97.50\%)}$
18(20 &6)	$\alpha _{1}$	-0.3553	0.5372	-1.3870	0.6201
	$\alpha _{2}$	0.4144	0.5797	-0.7269	1.5560
	$\alpha _{3}$	0.0363	0.5138	-0.9527	1.0400
	$\alpha _{4}$	0.9047	0.5448	-0.1325	2.0140
	$\alpha _{5}$	0.7546	0.5100	-0.2522	1.7900
	$\alpha _{6}$	-0.2815	0.3920	-1.0060	0.5173
	$\alpha _{7}$	0.4952	0.2707	-0.0085	1.0270
	$\gamma$	-0.1025	0.1734	-0.4249	0.2390
	$\psi$	-1.7320	0.4147	-2.5520	-0.9451
	$\vartheta$	-0.2822	0.1176	-0.5226	-0.0653
	$\xi$	-0.8246	0.1587	-1.1450	-0.5167
54(10)	$\alpha _{1}$	0.1051	0.2939	-0.4522	0.6736
	$\alpha _{2}$	-0.1363	0.2784	-0.6854	0.4119
	$\alpha _{3}$	0.2195	0.2881	-0.3424	0.7763
	$\alpha _{4}$	0.1937	0.2873	-0.3593	0.7665
	$\alpha _{5}$	0.1010	0.2921	-0.4732	0.6801
	$\alpha _{6}$	0.4376	0.1256	0.1903	0.6834
	$\alpha _{7}$	0.5031	0.1121	0.2888	0.7236
	$\gamma$	-0.1627	0.1068	-0.3688	0.0468
	$\psi$	-1.1910	0.2371	-1.6420	-0.7255
	$\vartheta$	-0.0887	0.0470	-0.1835	0.0018
	$\xi$	-0.9061	0.0937	-1.0930	-0.7318
180(5 &3)	$\alpha _{1}$	-0.0121	0.2217	-0.4340	0.4203
	$\alpha _{2}$	-0.0042	0.2247	-0.4505	0.4319
	$\alpha _{3}$	0.3473	0.2343	-0.1066	0.8064
	$\alpha _{4}$	0.4331	0.2350	-0.0284	0.8947
	$\alpha _{5}$	0.3283	0.2293	-0.1188	0.7739
	$\alpha _{6}$	0.1849	0.0936	0.0023	0.3670
	$\alpha _{7}$	0.5730	0.1032	0.3746	0.7754
	$\gamma$	-0.0852	0.0941	-0.2701	0.0956
	$\psi$	-0.7435	0.2031	-1.1410	-0.3454
	$\vartheta$	-0.1052	0.0211	-0.1486	-0.0645
	$\xi$	-1.9430	0.0792	-2.0980	-1.7920

Fixing sample sizes and priors across scenarios and examining the effect of varying sequences on parameter estimates, a consistent trend in magnitude and direction of the estimates and their log-transformation were observed across all scenarios. For example, the parameter estimates of results obtained from the model when sample size and time sequence 54(10) and $54(20 \& 6)$, 18(10) and $18(5 \& 3)$, 180(10) and $180(5 \& 3)$ under informative prior scheme were not markedly different in terms of their magnitude and direction. As an example, the posterior means and standard deviations obtained for the model scenario, sample size and visit scheme 180(10) under informative prior scheme from were $\alpha _{1}:0.100(0.183)$, $\alpha _{2}:0.065(0.194)$, $\alpha _{3}:0.106(0.069)$, $\alpha _{4}:-0.036(0.137)$, $\alpha _{5}:0.226(0.138)$, $\alpha _{6}:0.348(0.0.058)$, $\alpha _{7}:0.724(0.068)$, $~\gamma :-0.023(0.0560)$, $\psi :-0.916(0.120)$, $\vartheta :-0.116(0.026)$, $\xi :-0.972(0.0.048)$. These estimates are not markedly different in magnitude and direction from when the time sequence changed to $20 \& 6$ under the same scenario where the resulting estimates obtained were $\alpha _{1}:0.200(0.133),\alpha _{2}:0.122(0.124),\alpha _{3}:0.335(0.125),\alpha _{4}:0.156(0.128),\alpha _{5}:0.149 (0.128), \alpha _{6}:0.302 (0.055), \alpha _{7}:0.779 (0.063), \gamma :-0.058 (0.050), \psi :-1.052 (0.114), \vartheta :-0.105 (0.024),\xi :-0.957 (0.044)$. This pattern was similarly observed across the other scenarios, fixing sample sizes, priors and varying the time-sequences and broadly demonstrates a consistency in estimation performance. This further indicates that varying time sequences do not considerably affect the resulting estimates. Examining the credible interval(CI) widths under the different schemes reveal an interesting trend. As the sample sizes across all scenarios increased, albeit keeping priors and time sequences constant, the CI widths were increasingly narrow, implying that when our proposed model is applied to datasets of increasing sample sizes, the resulting estimates are obtained with higher precision. For instance, as an example, we compare parameter estimates and their CI widths under a select Gaussian non-informative prior scenario for these model scenarios 18(10), 54(10) and 180(10) (see Table 11). The trend observed from the presented estimates are quite obvious; increasing sample sizes applied to the proposed Bayesian Bernoulli-Exponential model increases precision of the model estimates. This broadly cuts across all scenarios.

**Table 11:** Credible interval widths for selected scenarios for the Bernoulli-Exponential model

Prior Scenario	Confidence Interval Widths for Scenarios			
	Parameter	18(10)	54(10)	180(10)
Non-informative prior	$\alpha _{1}$	2.5230	1.2630	0.5893
	$\alpha _{2}$	2.5233	1.1412	0.5893
	$\alpha _{3}$	2.2581	1.1670	0.5789
	$\alpha _{4}$	2.2239	1.1355	0.5818
	$\alpha _{5}$	2.0500	1.3101	0.6033
	$\alpha _{6}$	1.6099	0.5066	0.2313
	$\alpha _{7}$	1.1259	0.5338	0.2711
	$\gamma$	0.7179	0.4332	0.2168
	$\psi$	1.6386	1.0169	0.9782
	$\vartheta$	0.3937	0.2397	0.1097
	$\xi$	0.6261	0.3849	0.1878

### Simulation results: evaluation of the Bayesian Bernoulli-Exponential model

**Fig. 1:**
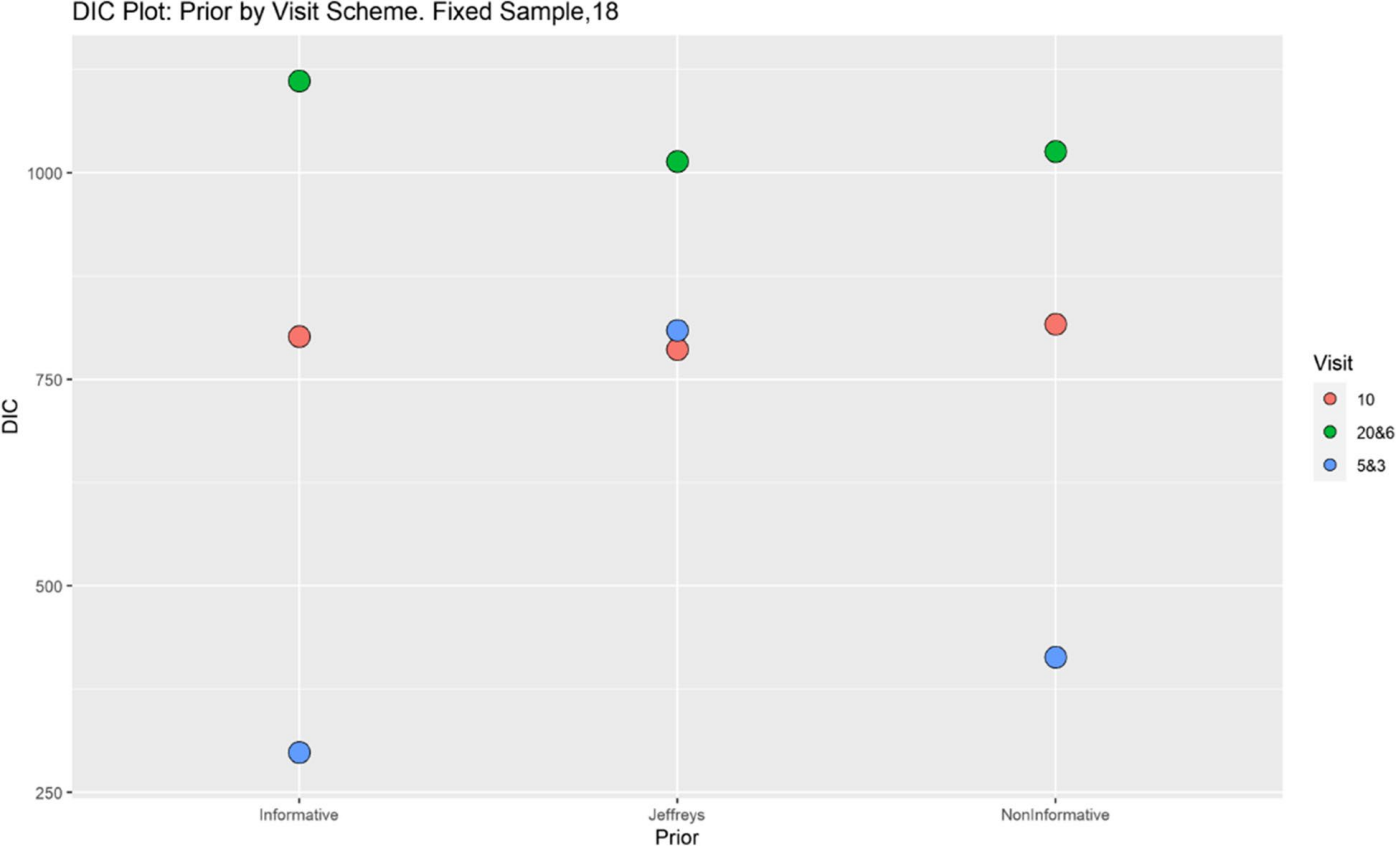
Deviance information criterion plot for keeping sample sizes fixed at 18 and examining influence across priors and design schemes

**Fig. 2:**
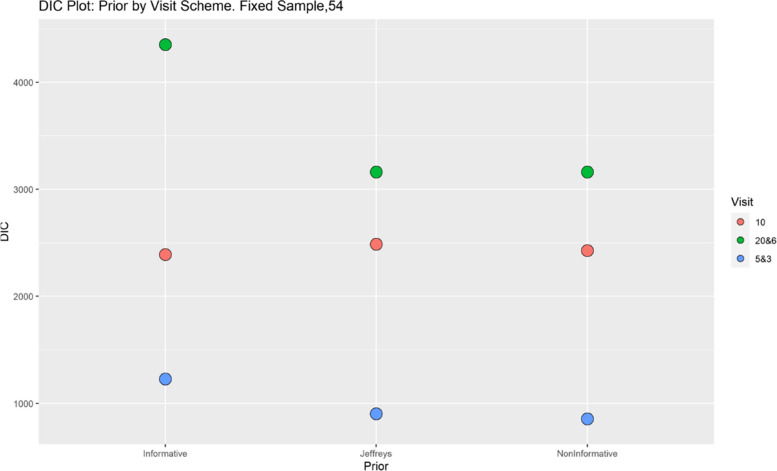
Deviance information criterion plot for keeping sample sizes fixed at 54 and examining influence across priors and design schemes

**Fig. 3:**
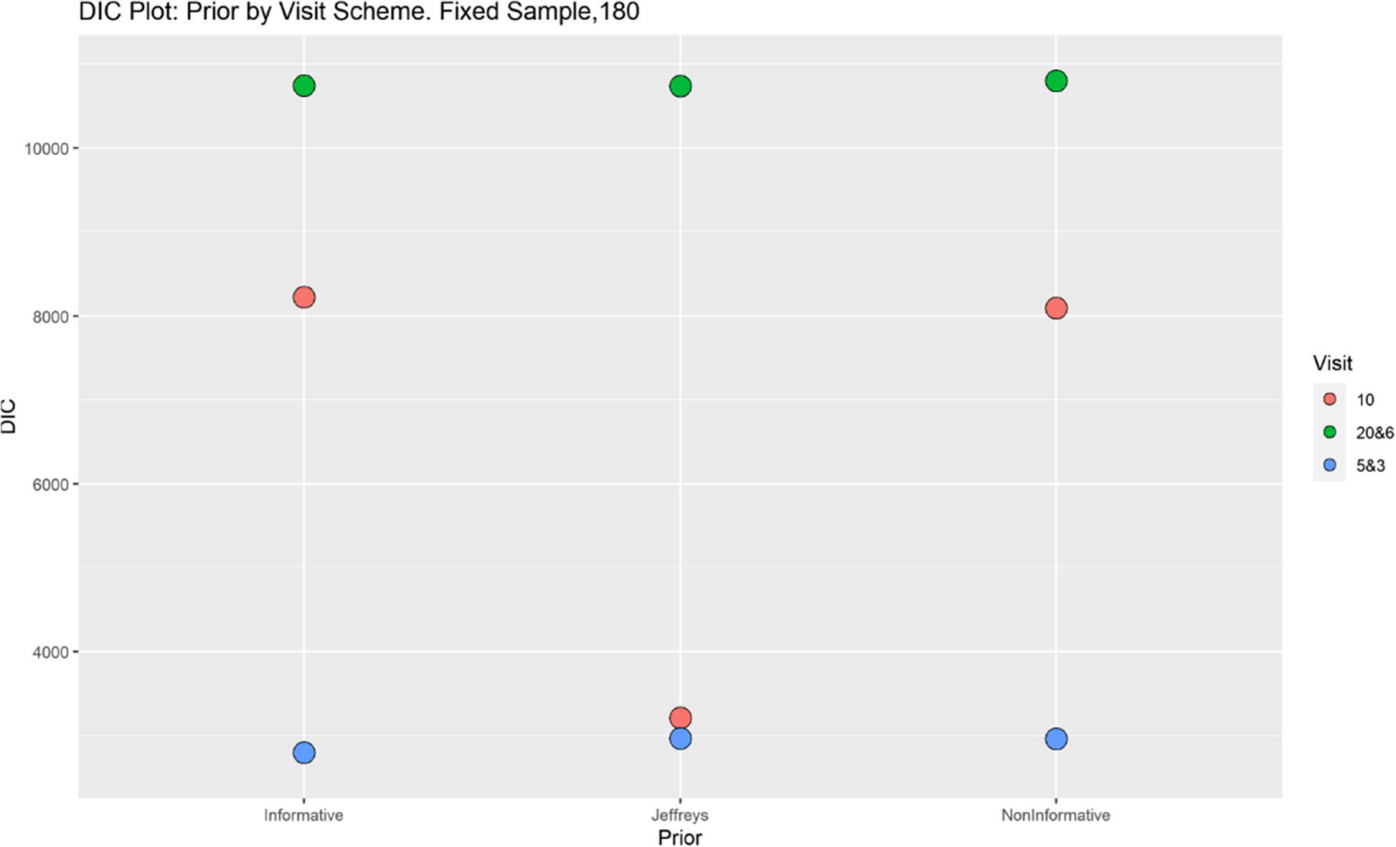
Deviance information criterion plot for keeping sample sizes fixed at 180 and examining influence across priors and design schemes

**Fig. 4:**
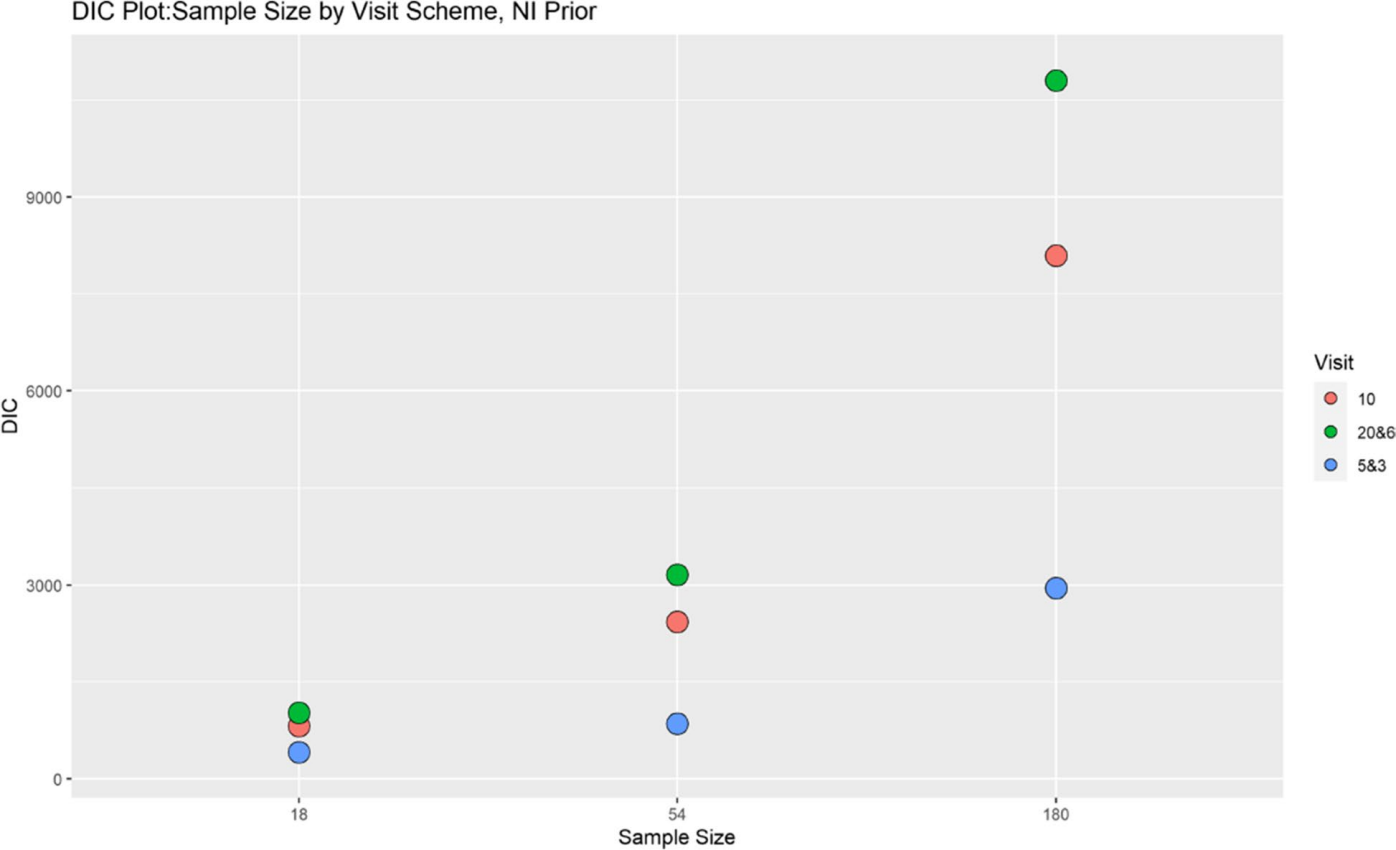
DIC plot for keeping prior fixed at gaussian non-informative and examining influence across sample size and design schemes

**Fig. 5:**
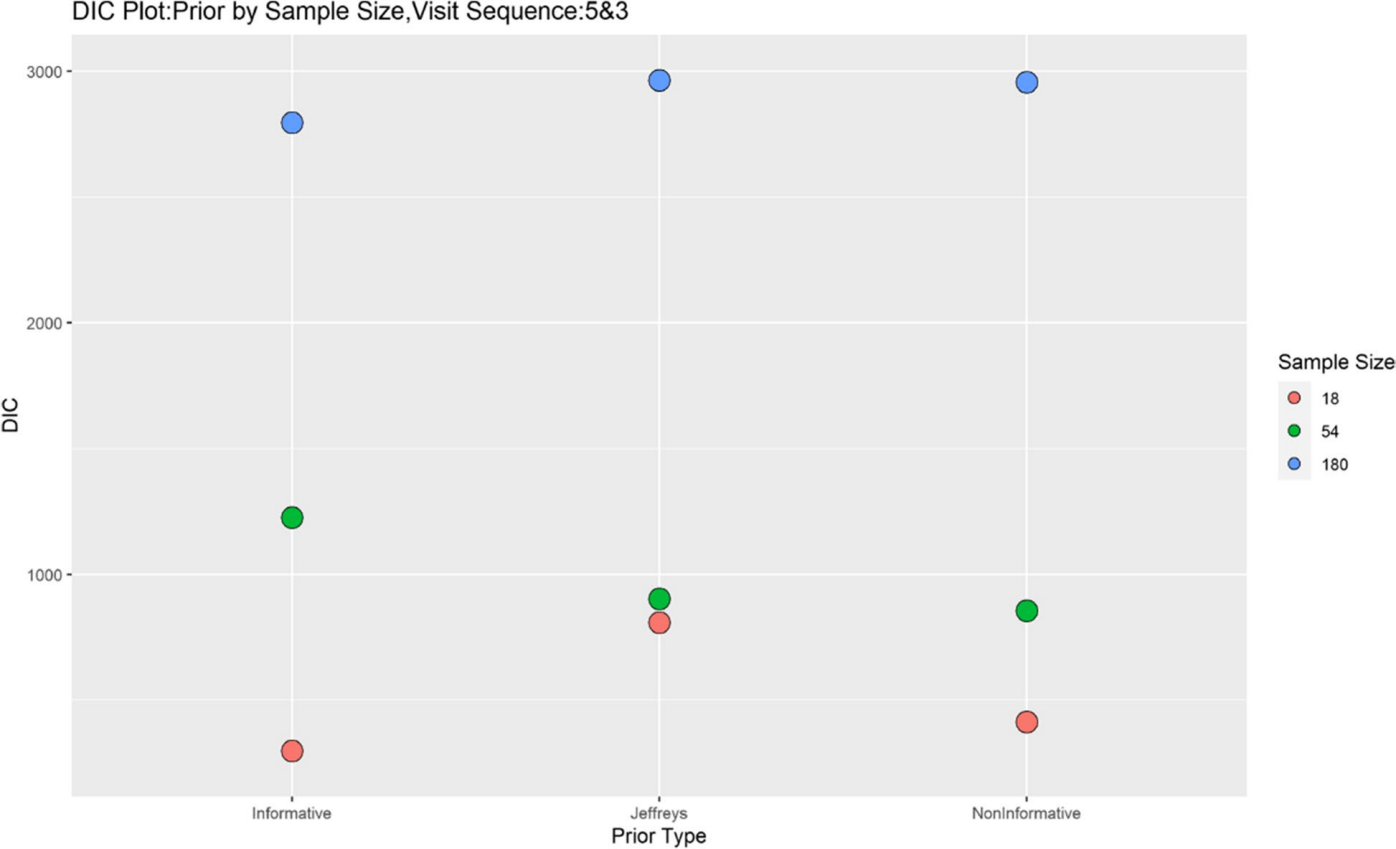
Deviance information criterion plot for keeping visit sequence fixed at $5 \& 3$ and examining influence across sample size and prior schemes

**Fig. 6:**
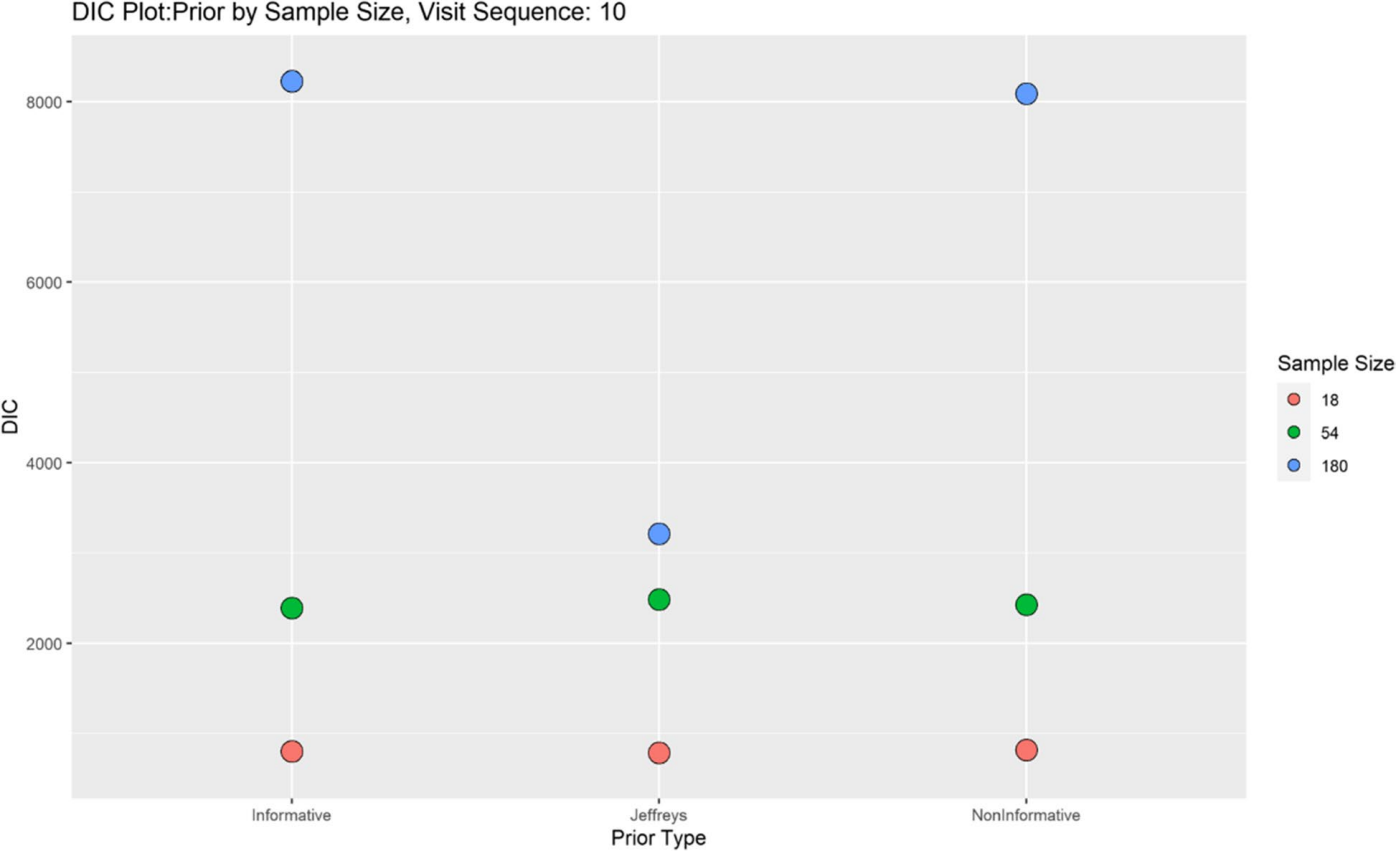
DIC plot for keeping visit sequence fixed at 10 and examining influence across sample size and prior schemes

**Fig. 7:**
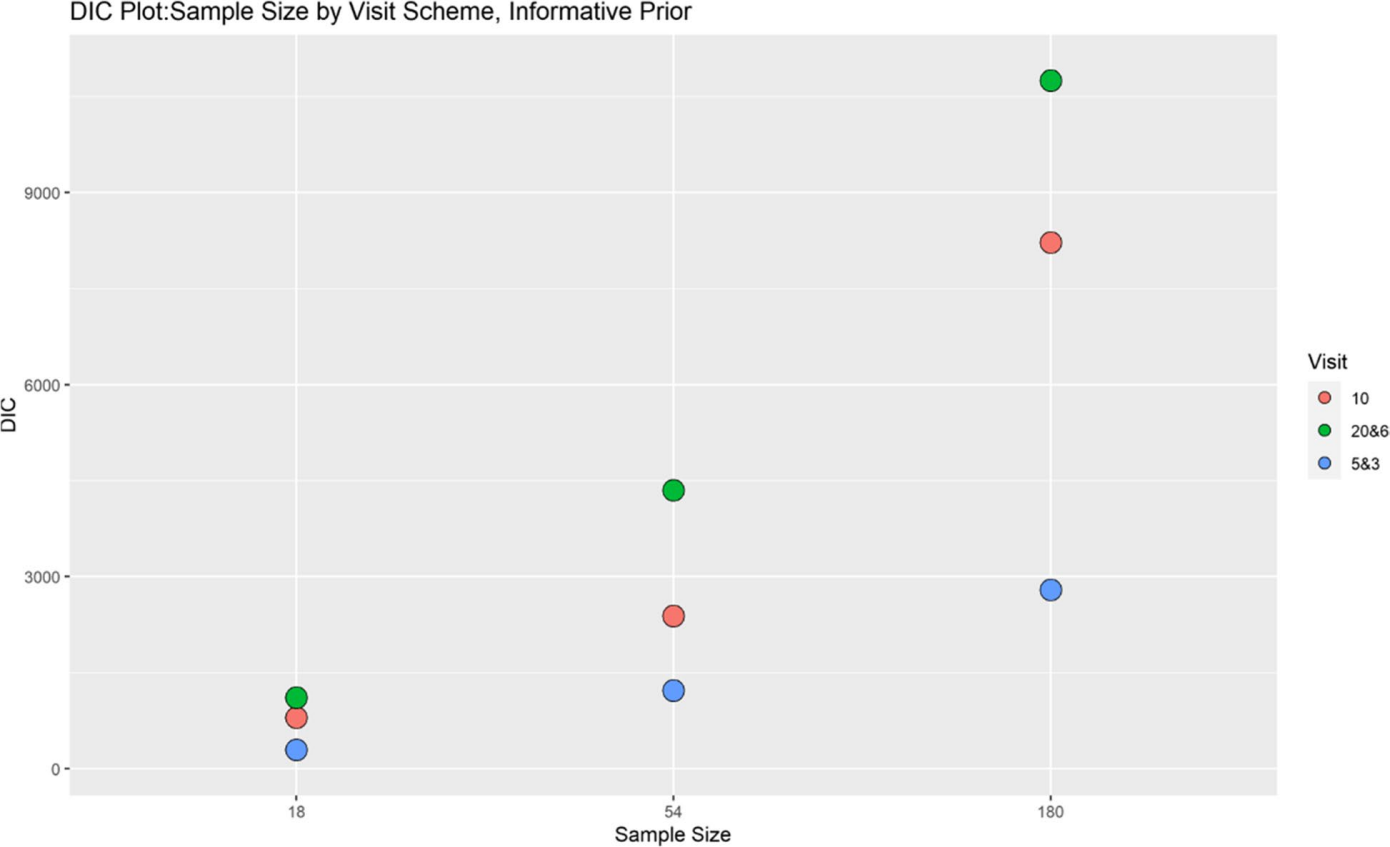
Deviance information criterion plot for keeping prior fixed at gaussian informative and examining influence across sample size and design schemes

**Fig. 8:**
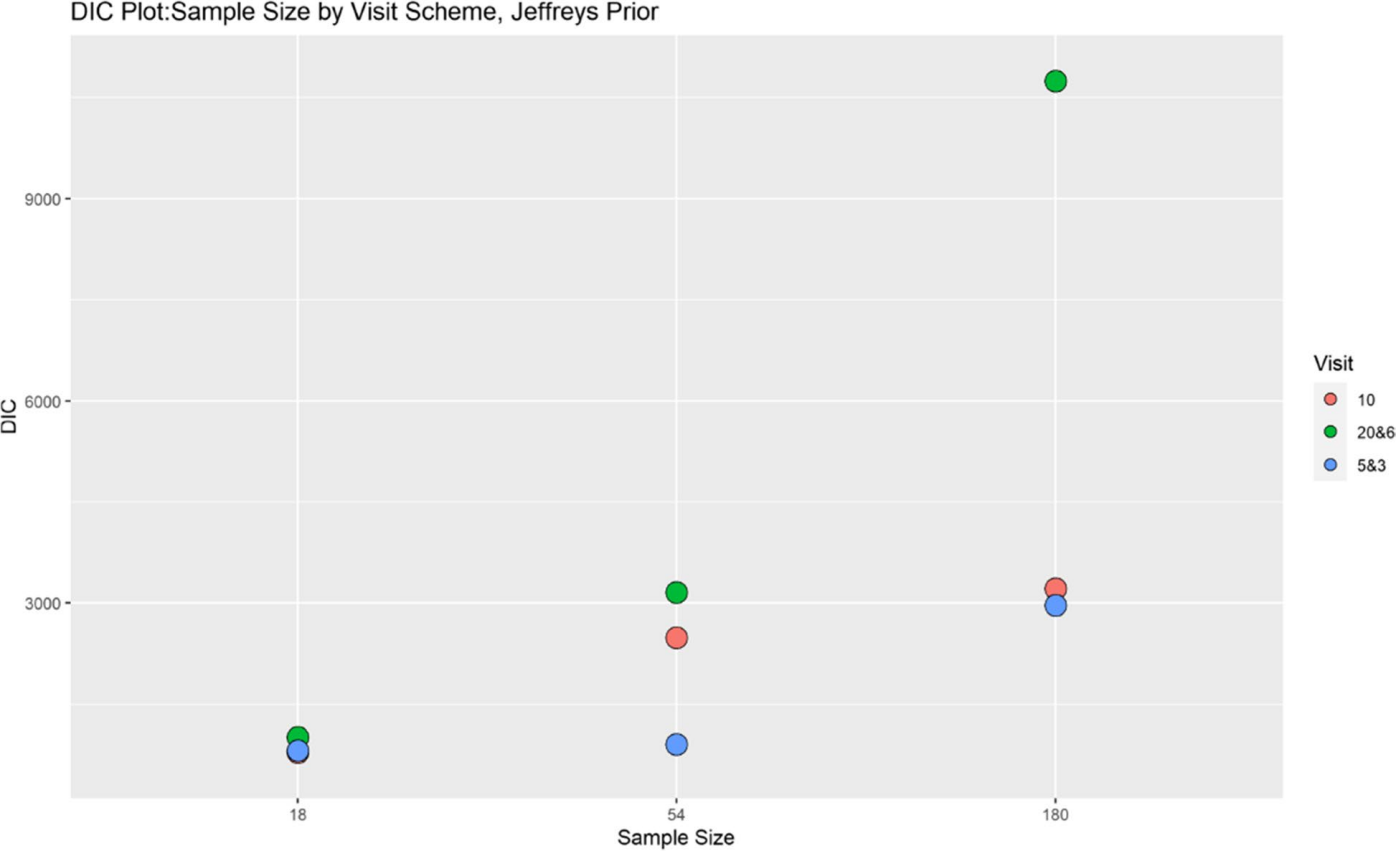
DIC plot for keeping prior fixed at jeffreys non-informative and examining influence across sample size and design schemes

Finally, model performance is evaluated under the various simulation scenarios via the Deviance Information Criterion (DIC). Since there are a lot of DIC values computed for varying scenarios, they are presented graphically for ease of evaluation and clarity. The DIC plots of the selected simulation scenarios applied to the model are presented in Figs. 1, 2, 3, 4, 5, 6, 7 and 8. First, we fix sample sizes and compare how the model performs across the type of prior and visit sequence. Regardless of the kind of prior chosen for the model parameters, it is observed in Fig. 1 that in the smallest sample considered, 18, the model performs better for the time sequence $5 \& 3$, reflected by lower DIC values across all prior scenarios. This is followed by the balanced time sequence, 10. In fact, there’s no marked difference between the DIC value of the time sequence $5 \& 3 (599.8)$ and 10(628.4) when considering the Jeffreys prior and fixing the sample size at 18. This trend is consistently observed, even when the sample sizes are fixed at 54 and 180 (see Figs. 2 and 3). The model still performs better for the time sequence $5 \& 3$ followed by 10. The next step in the model evaluation process involved fixing priors and comparing the models across competing sample sizes and sequences. For both Gaussian informative and non-informative priors, the DIC’s are very large for the time sequence $20 \& 6$ and sample size 180 signaling that the model may not be robust for scenarios where visit sequences of individuals vary significantly. When Jeffreys prior is considered, yet again DIC’s obtained for the model in small sample size 18 and sequence type $5 \& 3$ are very low indicating better performance followed closely by sample size 54, time sequence $5 \& 3$. This model scenario performs better across all samples and sequences than the considered Informative and Non-Informative Prior Scenario. The DIC values were at par in samples 54 and 180 for time sequence 10 and $5 \& 3$ when the Jeffreys prior was considered. Finally, an observation of model performance across sample size and prior schemes while keeping the time sequence fixed is made. Across time sequence $5 \& 3$, the model performs better overall for sample size 18 and 54 regardless of prior chosen. No marked differences are observed however when the Jeffreys prior is used for sample size 18 and 54 as evidenced by Fig. 5. Furthermore, model performance does not broadly vary for the sample size 180, regardless of the prior chosen for sequence $5 \& 3$ and $20 \& 6$. The results for the visit sequence 10 are quite consistent with $5 \& 3$ when compared. Models perform better in small sample size 18 scenarios as reflected by their lower DIC values, followed by 54.

The DIC values for sample size 54 and 180, however are close when the Jeffreys prior is considered for time sequence 10. Overall, model evaluation of the Bayesian Bernoulli-Exponential Model suggest a relatively better fit for small and medium sample size scenarios (18 and 54) with less varying time sequences (5 &3) and (10), regardless of prior choice. For larger samples (180), the models performs fairly well for less varying time sequences (5 &3) but not significantly so for time sequences (20& 6) regardless of the choice of prior.

### A model application to bladder cancer recurrence data

In this section, the proposed Bayesian Joint Bernoulli-Exponential model is applied to a real-world dataset, called the Bladder Cancer Data. This data is openly available in $\text{R}$ software, specifically in the “Survival” package [36] and results from a clinical trial on patients with bladder cancer conducted by the Veterans Administration Co-operative Urological Research Group (VACURG) [10, 11]. The bladder cancer dataset in $\text{R}$ software comprises information on 85 subjects, measured four times, with randomly assigned treatments of only thiotepa or a placebo. 38 patients are assigned to the placebo group and 47 to the treatment(thiotepa) group. Data on patient experienced number of recurrences are collected including the number of initial tumours present pre-trial randomization. Other variables include “stop”, which measures the time interval in months since the last visit. The next scheduled visit is dependent on bladder tumor recurrence at the time of measurement, indicating that time can be considered informative, and that subsequent visits are likely be influenced by previous visits. Also, the intensity of visits depend on tumor recurrences. Furthermore, there is an “event” variable, which is a binary variable representing the recurrence of tumor(1) or (0) for non-recurrence attributable to reasons like death. The variables along with their description are given in Table 12 below.

**Table 12:** The bladder cancer data (called bladder) in R software

Variable	Description
ID	Patient id
Treatment Type(rx):	1=Placebo 2=Thiotepa
Number:	Initial number of tumours (8=8 or more)
Tumor size:	Size in (cm) of largest initial tumour
Stop:	Recurrence or censoring time
Enum:	Which recurrence (up to 4)
Event Status:	1=Recurrence 0= Other Status(Can include death for any reason)

This data is analyzed with the following objectives in mind. Is there an effect of treatment type, size in centimeters(cm) of the largest initial tumor, initial number of tumors on the likelihood of tumor recurrence? Furthermore, is there an effect of prior recurrences(outcomes) on the likelihood of current recurrence? To answer these research questions, our proposed Bayesian Bernoulli-Exponential Joint model is fitted to the data. The binary “event” variable is used as the response and the predictors included in the model are treatment type, size in cm of the largest initial tumor, initial number of tumors and other time variables. Just as previously discussed in the Data and methods section, the Bayesian model involves the specification of a joint likelihood, priors and then the posterior distribution.

Here, three types of priors are considered and compared across the models. In this regard, the non-informative Gaussian priors considered for this model is,

$$\begin{aligned} p(\varvec{\alpha })&\sim N\left( \varvec{\text{0}}_{s}, 10^{8}\varvec{\text{I}}_{s}\right) \\ p({\gamma })&\sim N\left( 0, 10^{8}\right) \\ p(\vartheta )&\sim N\left( 0, 10^{8}\right) \\ p(\psi )&\sim N\left( 0, 10^{8}\right) \\ p(\xi )&\sim N\left( 0, 10^{8}\right) \end{aligned}$$

The Gaussian Informative priors considered for this model is,

$$\begin{aligned} p(\varvec{\alpha })&\sim N\left( \varvec{0.4}_{s}, 4.0\varvec{\text{I}}_{s}\right) \\ p({\gamma })&\sim N\left( 0.2, 0.1\right) \\ p(\vartheta )&\sim N\left( 0.5, 0.5\right) \\ p(\psi )&\sim N\left( 0.2, 0.2\right) \\ p(\xi )&\sim N\left( 2.0, 0.2\right) \end{aligned}$$

Furthermore, we consider Jeffreys non-informative priors for the $\alpha$ parameters and Gaussian non-informative priors for the visit parameters. The resulting posterior distribution of the Bayesian Bernoulli-Exponential Joint model for the bladder cancer data, for the instance where the Jeffreys prior considered for the parameters of the Bernoulli response process and Gaussian priors for the visit parameters in non informative settings is considered is;

19 $$\begin{aligned} p(\varvec{\alpha }, \vartheta ,\psi ,\xi ,\phi |\varvec{Y}_{i},\varvec{t}_{i},\varvec{X} )=L\left( \Theta , y_{1},y_{2},y_{3} \cdots , y_{s}\right) \times p(\varvec{\alpha }|\phi )\times p(\vartheta )\times p(\psi )\times p(\xi ) \end{aligned}$$

$$\begin{aligned}{}&=\prod _{i=1}^{s}\Bigg \{\exp \bigg \{y_{i1} \ln \left( \frac{\mu _{i1}}{1-\mu _{i1}}\right) +\ln (1-\mu _{i1})\bigg \}\times \prod _{k=2}^{n_{i}} \exp \Big \{y_{ik} \ln \left( \frac{\mu _{ik}}{1-\mu _{ik}}\right) \\ {} &\quad+\ln (1-\mu _{ik})\times \exp \left( \xi +\gamma y_{ik-1}\right) \times \exp \left( -\exp \left( \xi +\gamma y_{ik-1}\right) t_{ik}\right) \Big \}\Bigg \}\\&\quad\times \left| \varvec{X}^{\prime } \varvec{P} \varvec{V}(\varvec{\alpha }) \Delta ^{2}(\varvec{\alpha }) \varvec{X}\right| ^\frac{1}{2}\times \frac{1}{\sqrt{2 \pi \nu _{\vartheta }^{2}}} \exp \left( -\frac{1}{2}\left( \vartheta -\mu _{\vartheta }\right) ^{2}\right) \times \frac{1}{\sqrt{2 \pi \nu _{\psi }^{2}}} \exp \left( -\frac{1}{2}\left( \psi -\mu _{\psi }\right) ^{2}\right) \\&\quad\times \frac{1}{\sqrt{2 \pi \nu _{\xi }^{2}}} \exp \left( -\frac{1}{2}\left( \xi -\mu _{\xi }\right) ^{2}\right) \end{aligned}$$

Here, $\varvec{V}(\varvec{\alpha })=\text {diag}\left( v_{1},v_{2} \ldots , v_{n}\right)$ and $v_{i}=\mu _{ik}(1-\mu _{ik})$. and,

- $\alpha _{s}$ are regression parameters representing the effect of the predictors; treatment type($x_{2}$), initial number of tumors, ($x_{3}$) and size in (cm)($x_{4}$) of the largest initial tumor on the likelihood of tumor recurrence.
- $\psi$ represents the effect of the prior recurrence on the mean response of the current recurrence and $\vartheta$ characterizes the effect of current recurrence time on the mean recurrence,
- $\xi$ is a constant parameter associated with time and $\gamma$ is the effect of the previous recurrence on the mean time.

Other components are already explained thoroughly in the Data and methods section. Note that the posterior distribution changes when the priors change in the Gaussian and non-Gaussian settings considered for all parameters. Then, after the posterior specification, we proceed with the joint parameter estimation with the Gibbs sampling approach in R software. For each of the three prior scenarios considered, the Markov chains are run iteratively 30,000 times, and the first 10,000 iterations are discarded to serve as burn-in. Convergence of the markov chains and associated posterior parameters are monitored via the Heidelberger and Welch tests. Then, posterior summaries are computed. Parameter significance is inferred via credible intervals and the models are compared with the Deviance Information Criteria Measure. Results of the Heidelberg and Welch convergence tests from the application to the bladder cancer data with the different prior scenarios are presented in Table 13. Inferring from the tests conducted, no issues were observed with the convergence of the MCMC chains. Overall, we can proceed with posterior summary inference with precision since the MCMC chains are in a stationary distribution.

**Table 13:** Heidelberger and welch test for the Bayesian Bernoulli-Exponential model for the bladder cancer data including three prior scenarios

Parameter	Stationarity Test	*P*-value	Halfwidth Test	Mean	Halfwidth
**Informative Prior Scenario**					
$\alpha _{1}$	passed	0.499	passed	0.222	0.019
$\alpha _{2}$	passed	0.195	passed	0.402	0.010
$\alpha _{3}$	passed	0.794	passed	0.006	0.003
$\alpha _{4}$	passed	0.462	passed	-0.049	0.006
$\gamma$	passed	0.503	passed	-0.118	0.006
$\psi$	passed	0.666	passed	-0.008	0.007
$\vartheta$	passed	0.207	passed	0.000	0.003
$\xi$	passed	0.618	passed	-0.127	0.004
**Non- Informative Prior Scenario**					
$\alpha _{1}$	passed	0.104	passed	-0.339	0.054
$\alpha _{2}$	passed	0.089	passed	0.466	0.666
$\alpha _{3}$	passed	0.555	passed	0.107	0.010
$\alpha _{4}$	passed	0.077	passed	0.189	0.020
$\gamma$	passed	0.542	passed	-0.021	0.013
$\psi$	passed	0.322	passed	0.106	0.011
$\vartheta$	passed	0.718	passed	0.095	0.008
$\xi$	passed	0.509	passed	-0.182	0.009
**Jeffreys Non-Informative Prior Scenario**					
$\alpha _{1}$	passed	0.134	passed	0.138	0.002
$\alpha _{2}$	passed	0.433	passed	2.005	0.016
$\alpha _{3}$	passed	0.083	passed	0.160	0.001
$\alpha _{4}$	passed	0.053	passed	0.061	0.001
$\gamma$	passed	0.613	passed	-0.012	0.008
$\psi$	passed	0.168	passed	0.416	0.011
$\vartheta$	passed	0.270	passed	0.080	0.008
$\xi$	passed	0.224	passed	-0.156	0.005

After convergence assessment of the model, inference based on the posterior summary measures is the next step. Posterior means, standard deviations and associated credible intervals of the prior scenarios are presented in Table 14 along with their corresponding DIC’s. The best model is chosen based on the least DIC value. Observing the results, the model under the Jeffreys non-informative prior, yielded the least DIC (1108) value. Ergo, parameter inference is based on the Bayesian Bernoulli-Exponential model with Jeffreys prior specified. The results demonstrate that the effect of treatment type is statistically significant on the likelihood of cancer recurrence inferring from its credible interval $\alpha _{2}=0.216$ (0.232, 0.411). The initial number of tumors have a significant effect $\alpha _{3}=0.036$ (0.001, 0.108) on the likelihood of cancer recurrence and hence a significant prognostic factor. Furthermore, the size in cm of the largest tumor has a significant marker on the likelihood of cancer recurrence. Afterwards, the time parameters are observed. The effect of prior tumor recurrence on the mean response of current tumor recurrence, represented by $\psi$ is statistically significant $-0.408(-1.009,-0.135)$, indicating that previous tumor recurrences influence the probability of subsequent recurrences. Additionally, the effect of current recurrence time($\vartheta$) is significant on average recurrence, reflected by the estimated probability (0.157)(0.018, 0.337).

**Table 14:** Results of the Bayesian Bernoulli-Exponential model applied to the bladder cancer data with different prior scenarios considered

**Gaussian Informative Prior Estimates**					**Deviance Information of Model**			
Parameter	Mean	SD	2.50%	97.50%	Dbar	Dhat	DIC	pD
$\alpha _{1}$	0.621	0.263	0.113	1.136	1119	1113	1126	6.652
$\alpha _{2}$	0.404	0.496	-0.566	1.374				
$\alpha _{3}$	-0.021	0.061	-0.140	0.100				
$\alpha _{4}$	-0.115	0.095	-0.300	0.068				
$\gamma$	0.165	0.126	-0.078	0.415				
$\psi$	-0.318	0.231	-1.229	-0.132				
$\vartheta$	0.070	0.090	-0.109	0.248				
$\xi$	-0.366	0.094	-0.550	-0.187				
**Gaussian Non-Informative Prior Estimates**					**Deviance Information of Model**			
Parameter	Mean	SD	0.025	0.975	Dbar	Dhat	DIC	pD
$\alpha _{1}$	0.256	0.312	-0.321	0.867	1114	1107	1121	6.953
$\alpha _{2}$	0.228	0.127	0.235	0.412				
$\alpha _{3}$	0.008	0.065	-0.118	0.135				
$\alpha _{4}$	-0.006	0.107	-0.206	0.198				
$\gamma$	-0.100	0.128	-0.352	0.150				
$\psi$	- 0.608	0.240	-1.079	-0.137				
$\vartheta$	-0.099	0.094	-0.287	0.081				
$\xi$	-0.216	0.095	-0.407	-0.036				
**Jeffreys Non-Informative Prior Estimates**					**Deviance Information of Model**			
Parameter	Mean	SD	0.025	0.975	Dbar	Dhat	DIC	pD
$\alpha _{1}$	0.174	0.123	0.007	0.457	1103	1098	1108	4.972
$\alpha _{2}$	0.216	0.125	0.232	0.411				
$\alpha _{3}$	0.036	0.029	0.001	0.108				
$\alpha _{4}$	0.048	0.039	0.001	0.145				
$\gamma$	0.055	0.129	-0.199	0.305				
$\psi$	-0.408	0.241	-1.009	-0.135				
$\vartheta$	0.157	0.091	0.018	0.337				
$\xi$	-0.287	0.096	-0.476	-0.101				

## Discussions and conclusions

Broad assumptions underlie the usage of longitudinal analysis approaches, ranging from univariate designs to the even the most complex conditional and marginal modeling approaches. One of the common assumptions, albeit implausible in certain scenarios, is the supposition that time is always fixed and predetermined by statistical design. Phenomenons may alter the time trajectory of study subjects, like sickness or adverse events in clinical trials, which may result in not only irregular time points for subjects, but also imbalanced data and differing visit intensities. This implies current visit outcomes being informative to subsequent ones. It is also important to emphasize that the issue of informative censoring may be less problematic in the context of an informative time/schedule designs, given the assumed observation schedule protocols. In simpler terms, individuals with more severe conditions requiring early interventions or treatments, which could lead to informative censoring, would also have shorter observation schedules and, consequently, more “frequent” measurements. This assumption underlies the simulation design for this study. In this article, we have developed a Bayesian joint model for longitudinal outcomes from the exponential family of distributions with particular emphasis on Bernoulli distributed longitudinal outcomes and exponentially distributed informative time points. An assessment of the influence of controlled sample size scenarios, visit and prior specification schemes on the estimated parameters of the proposed Bayesian Bernoulli-Exponential joint model was performed via simulations and was evaluated based on Deviance Information Criteria.

The methods commenced with specifying likelihoods for the joint outcome and time distributions, specification of priors, and then a discussion on the Markov Chain Monte Carlo Approach for estimating posterior parameters. The priors considered were Gaussian informative priors, Gaussian non-informative priors and Jeffreys non-informative priors. Convergence analysis was performed with the Heilderberg and Welch Test. Once the models converged, posterior inference followed and models were evaluated based on Deviance Information Criteria. Inference from the Heidelberger and Welch Tests conducted across selected simulation scenarios for the Bayesian Bernoulli-Exponential broadly suggested no pertinent issues with the convergence or stationarity of MCMC chains for estimated parameters irrespective of prior specified, sample size or visit schemes. Fixing sample sizes and priors across selected scenarios of the model and examining effect of varying sequences on parameter estimates, a consistent trend in magnitude and direction of the estimates and their transformations were observed.

As sample sizes increased, albeit keeping priors and time sequences constant, credible interval widths were increasingly narrow, indicating that when the proposed model is applied to datasets of increasing sample sizes, resulting estimates are obtained with higher precision. Overall, evaluation made for the Bayesian Bernoulli-Exponential model indicated better performance for the less intense visit sequence $5 \& 3$ scenario, reflected by lower DIC values, followed by the balanced visit sequence 10 regardless of sample size or prior type. Sample sizes across various simulation scenarios performed similarly well, only that the difference in performance was largely attributable to the sequence of individual visits. Finally, the proposed model has been applied to a bladder cancer recurrence data to serve as an application example.

## Data Availability

Beyond the simulation analysis, the data that support the findings of this study and for the model application is openly available in R software, called ‘bladder’ specifically in the “Survival” package [36] and results from a clinical trial on patients with bladder cancer conducted by the Veterans Administration Co-operative Urological Research Group (VACURG) [10, 11].
